# pH-Responsive Gallol-Functionalized Hyaluronic Acid-Based Tissue Adhesive Hydrogels for Injection and Three-Dimensional Bioprinting

**DOI:** 10.1021/acsami.3c02961

**Published:** 2023-07-06

**Authors:** Hatai Jongprasitkul, Vijay Singh Parihar, Sanna Turunen, Minna Kellomäki

**Affiliations:** † Biomaterials and Tissue Engineering Group, BioMediTech, Faculty of Medicine and Health Technology, Tampere University, 33720 Tampere, Finland; ‡ Brinter Ltd, 20520 Turku, Finland

**Keywords:** hyaluronic acid, gallic acid, pH-responsive, bioprinting, bioink blend, photocrosslinking

## Abstract

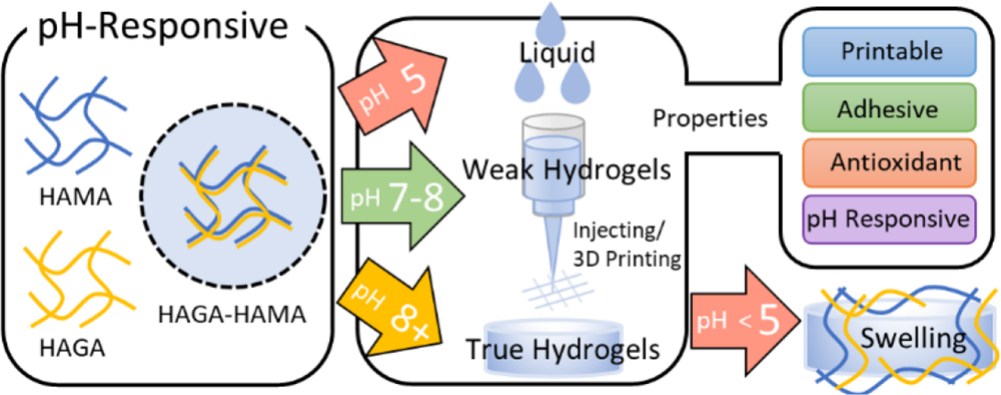

The major challenges of hyaluronic acid-based bioinks in extrusion-based three-dimensional bioprinting are poor printability and low printing accuracy. To tackle the challenges, we developed a bioink in which two components are blended: gallic acid-functionalized hyaluronic acid (HAGA) and hyaluronic acid methacrylate (HAMA). In the precursor phase, the blend’s HAGA component enables pH-dependent viscosity modulation that results in improved injectability and printability at physiological temperature. Postprinting, the blend’s HAMA component is photocrosslinked to create a true hydrogel with a complementary network of both HAGA and HAMA. The ready structures of the HAGA-HAMA hydrogel showed sufficient printing quality and accuracy compared to plain HAMA. The blend also displayed enhanced viscoelastic properties and stable swelling behavior. In addition to the pH tunability, the HAGA component also imparted tissue adhesion and antioxidant activity. This bioink has the potential to be printed directly on an infected wound site due to its adhesiveness to tissue and dimensional stability in situ.

## Introduction

Hyaluronic acid-based (HA) hydrogels have been considered an attractive choice for bioinks. The various reactive functional groups allow HA hydrogels to be chemically modified by the conjugation of biorthogonal moieties or bioactive molecules.^1,2^ Modification with methacrylate (MA) groups is the most common way to obtain highly versatile bioinks and a hydrogel network can be formed via photopolymerization reaction.^3−5^ Recently, hyaluronic acid methacrylate (HAMA), with a high degree of MA-modification, has been used for light-based three-dimensional (3D) bioprinting, such as stereolithographic and digital light processing.^6^ However, the printing of HAMA using an extrusion-based 3D bioprinter remains challenging due to its low mechanical properties, poor printability, and poor printing accuracy.^7^ HAMA’s printability can be improved by blending it with high shear-thinning or stimuli-responsive precursors/hydrogels to create a complementary network, which can compensate for the HAMA’s insufficient properties.^8−13^ Over the past decades, the development of tissue adhesive hydrogels has been reported with various techniques, including mussel-inspired chemistry and supramolecular interactions.^14^ However, the integration between high printability, stimuli-responsiveness, and tissue adhesion in one biomaterial ink is still challenging.

To improve the printability of bioinks, blending bioinks with high-molecular-weight polymers could be an alternative option. However, the physical blending of two different molecular weight polymers may create an immiscible mixture as blending requires compatibility of polymer properties.^15^ Furthermore, the biological functions might be disrupted because a higher pressure is required during the printing process.^16,17^ Precrosslinking techniques are an effective way to convert unprintable inks into printable ones capable of forming 3D constructs. Precrosslinking techniques create a weak hydrogel network, giving enough stability to sustain shape fidelity during the printing.^18^ Several precrosslinking approaches have been studied to improve the printability of bioinks for extrusion-based 3D bioprinting.^19^ The most common ways are to utilize ionic crosslinking (e.g., for alginate, gellan gum),^20−22^ enzymatic crosslinking (collagen),^23^ pH (chitosan),^24^ or temperature changes (gelatin).^25−27^

Stimuli-responsive hydrogels have also been investigated as candidates for bioinks and can be induced by exposing the ink to various environmental changes, including pH, temperature, light, and ions.^28^ These properties provide versatility to bioinks, as they harness the on-demand tunability of bioinks and can be used for various applications.^29^ The pH-responsive hydrogels have gained wide interest because of their excellent adaptation in physiological conditions for in situ bioprinting applications.^30^ Moreover, pH-stimuli can better control bioink’s stability in the defect site due to the different pH during healing stages.^31,32^ Only a few reports have explored the pH-responsive properties of bioinks to obtain printable hydrogels; for example, the pH-induced chitosan hydrogel was printed into a concentrated NaOH bath, forming the intramolecular–intermolecular hydrogen bonds.^24^

Moreover, an injectable hydrogel with self-healing and tissue adhesive properties is an interesting class of hydrogels. Self-healing injectable hydrogels can temporarily fluidize under shear stress and recover their original structure and mechanical properties after the release of the applied stress. This ability makes them easily injectable at the wound site. Additionally, as self-healing injectable hydrogels possess tissue adhesive properties, they can adhere effectively to the wound site and facilitate sutureless implantation of hydrogel constructs.^33^ Shin and Lee have reported the combination of gallol-tethered hyaluronic acid and oligo-epigallocatechin (OEGCG) gels with pH-dependent behavior for injectable hydrogels at basic conditions.^1^

In this work, as a novel component for bioink, we developed gallic acid-functionalized hyaluronic acid (HAGA) to establish pH-responsiveness that can control the printability of precursors as well as both the mechanical and swelling behavior of hydrogels. GA is a polyphenol compound with three phenol units known as catechol moieties and is also recognized for its tissue adhesive properties and antioxidant activity.^34^ We hypothesized that the precursor blend of HAGA-HAMA could achieve high printability and injectability at pH 7.5–8. The pH change serves as a precrosslinking method for the precursor during printing followed by UV postcrosslinking to stabilize the printed constructs. The precursor formulations could be printed without any additional viscosity enhancers. The HAGA component enhances pH responsiveness at basic pH, which results in an increase of tissue adhesion via phenolic group oxidation. This phenomenon mimics mussel adhesion due to the higher interaction of reductive cysteine-rich proteins.^35^

**Figure 1:**
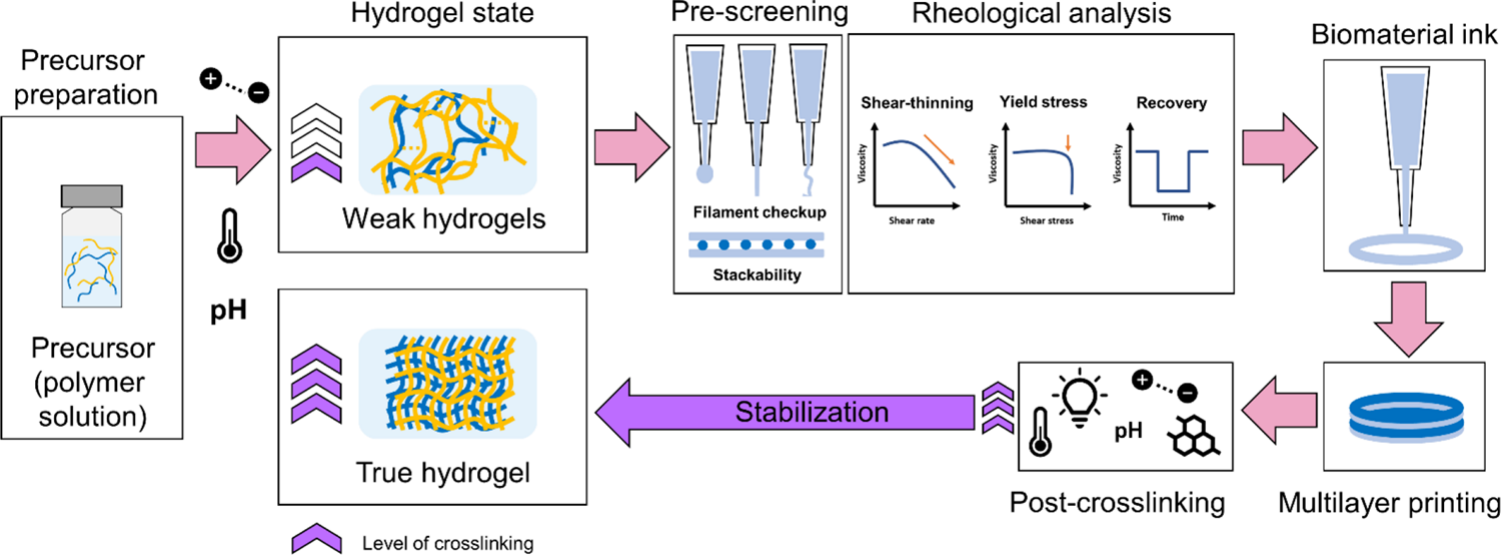
Flow chart demonstrates the process of biomaterial ink evaluation through the definitions of precursor, weak hydrogel, true hydrogel, and biomaterial ink. (precursor → weak hydrogel → biomaterial ink → true hydrogel). Precursor, polymer solution or pre-hydrogel solution without crosslinking. Weak hydrogel, weakly crosslinked hydrogel (extrudable). Biomaterial ink, printable precursor (weak hydrogel) or precursor candidate for 3D bioprinting that has been screened for printability through various evaluation steps: precursor preparation, precrosslinking, prescreening for printability (filament formation and stackability), rheological analysis (degree of shear-thinning, yield stress, and recovery behavior), 3D printing (multilayer printing), and postcrosslinking (stabilization). True hydrogel, crosslinked hydrogels with mechanically stable to maintain the structural integrity after printing.^36^

**Figure 2:**
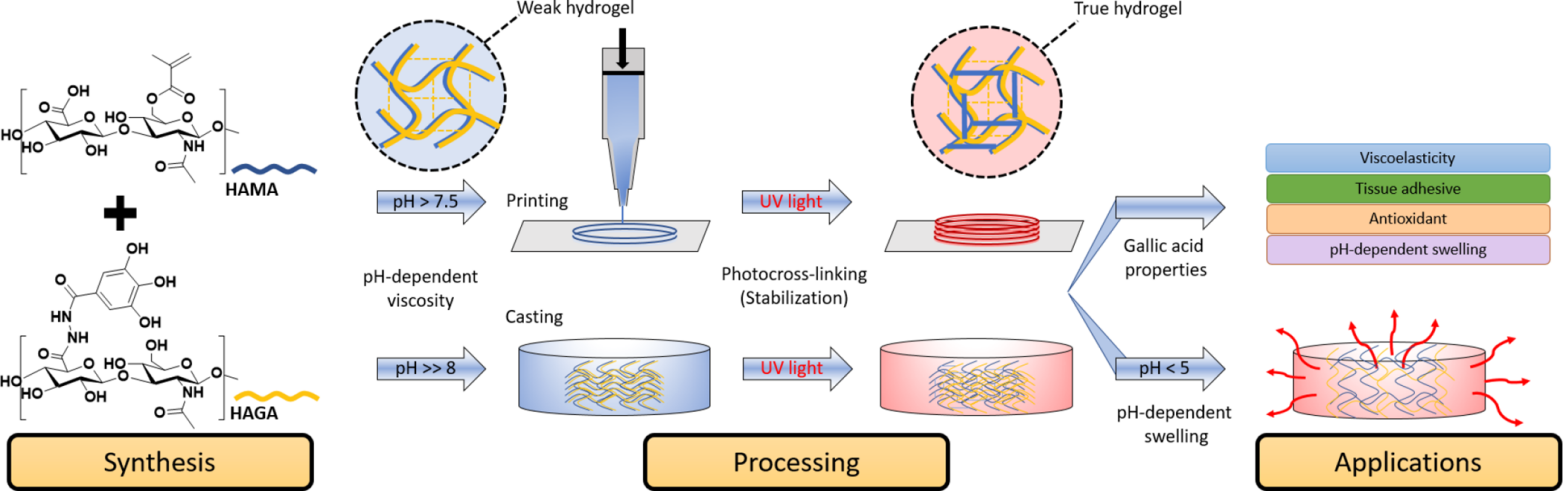
Schematics of HAGA and HAMA blend, combining the viscosity modulation of pH-dependent precursors for casting and extrusion-based 3D bioprinting. 3D printing of the complementary network hydrogel is done in two steps: first, the viscosity of the precursor is enhanced via pH change to obtain proper printability, described as “ink”, and next, photocrosslinking is used after printing. The GA-based hydrogels demonstrate viscoelasticity, tissue adhesion, and antioxidant and pH-dependent swelling behavior.

This article reports the synthesis of HAGA with 10 and 20% GA modification and HAMA with 15% methacrylation and their blending to a 1:1 volume ratio. The evaluation of biomaterial ink printability was addressed in this article through an evaluation process illustrated in Figure 1, which is based on our previous studies.^7,21,36^ The rheological characterization of the HAGA-HAMA precursor at different pH was performed. Furthermore, the effect of GA functionalization on the viscoelastic properties of hydrogels was investigated. Additionally, HAGA conjugation provided tissue-adhesive properties and antioxidant activity to the HAGA-HAMA hydrogel. The schematics in Figure 2 describe the entire process of synthesis, processing, and postprocessing of the HAGA-HAMA precursor. We also highlight that the pH-responsive precursors offer a flexible way to control the ink’s viscosity for printing. Furthermore, the viscoelastic properties and tissue adhesiveness of the photocrosslinked hydrogels can be easily modified by changing the pH and degree of GA modification.

## Materials and Methods

Hyaluronic acid (*M*_w_ 100 kDa) was purchased from LifeCore Biomedical (Chaska, USA). Methacrylic anhydride, gallic acid (3,4,5-trihydroxy benzoic acid), hydrazine hydrate, 1-ethyl-3-(3-dimethyl aminopropyl)-carbodiimide hydrochloride (EDC), 1–hydroxy benzotriazole hydrate (HOBt), dimethyl sulfoxide (DMSO), and Irgacure 2959 (I2959) were purchased from Merck KGaA, Darmstadt, Germany. Dialysis membranes used for purification were purchased from Spectra Por-6 (MWCO 3500). DI water (deionized water, Miele Aqua Purificator G 7795, Siemens) was used. Dulbecco’s phosphate-buffered saline (DPBS) was prepared in the lab. All solvents were of analytical quality. Nuclear magnetic resonance (NMR) analysis was carried out on an NMR spectrometer (Varian Mercury 300 MHz, Agilent Technologies, Inc., USA).

### Synthesis of Hyaluronic Acid Methacrylate

Methacrylated hyaluronic acid (HAMA) with ∼15% MA was prepared by adjusting the ratio of methacrylic anhydride in the reaction, as has been described previously in ref (7). In brief, 400 mg of sodium hyaluronate was dissolved in 100 mL of deionized water at pH 9. Next, methacrylic anhydride was added dropwise, providing the amount equal to the defined modification (500 μL). The reaction was carried out for 7 h at 4 °C while maintaining pH ∼ 8. After that, the reaction mixture was dialyzed with a 3.5 kDa MWCO membrane against deionized water for 72 h (2 × 2 L, 12 h) at RT. Thereafter, the solution was lyophilized, and the product was obtained. The MA in HAMA was quantified by 1H NMR. The measurement was performed at RT. The synthesis procedure of HAMA is displayed in Figure S-1.

### Synthesis of Gallic Acid-Functionalized Hyaluronic Acid

400 mg of HA (1 mmol of HA, in equivalent) was dissolved in 75 mL of DI water followed by the addition of 1 mmol N-hydroxy benzotriazole (HOBt, 153 mg, 1 equiv). The gallic acid hydrazide (Figure S-2) (GA-Hyd, 184 mg, 1 equiv) was separately dissolved in 25 mL of DMSO and added to the stirred reaction mixture solution dropwise and allowed additional stirring for 30 min. The pH of the reaction solution was adjusted to 4.75 using 1 M HCl and 1 M NaOH. For the 10 and 20% GA modification, 0.15 mmol (29 mg, 0.15 equiv) and 0.30 mmol (57.5 mg, 0.30 equiv) of EDC were added, respectively. The mixture was stirred overnight. The reaction mixture was then loaded into a dialysis bag (Spectra Por-6, MWCO 3500 g/mol) and dialyzed against dilute HCl (pH = 3.5) containing 100 mM NaCl (6 × 2 L, 48 h) and then dialyzed against deionized water (4 × 2 L, 24 h). The solution was lyophilized to obtain a white solid fluffy material. The conjugation of gallic acid and the degree of modification of gallic acid in the hyaluronic acid was further ascertained by the presence of distinctive aromatic peaks at 6.98 and 6.93 ppm of GA in the 1H NMR spectrum. The HAGA synthesis is displayed in Figure S-3.

### Preparation of pH-Responsive Precursors

All precursors were prepared at a concentration of 5% w/v in DPBS. The PI, Irgacure 2959, was added into the HAMA precursor at a concentration of 0.5% w/v. The precursors of HAGA and HAMA were mixed into a ratio of 1:1 with two formulations: HAGA10-HAMA15 and HAGA20-HAMA15. The pH of all precursor formulations was slowly adjusted using 0.5 M NaOH and varied into acidic (pH = 4 and 5), neutral (pH = 7), and basic (pH = 8 and 9) pH.

### pH-Dependent Rheological Behavior of Precursors

The rheological characterizations were carried out on a rheometer (Discovery HR-2, TA Instruments Inc., USA) using a plate-to-plate geometry with a diameter of 12 mm. Different formulations of precursor with different pH were measured using flow mode. The measurements were made at 37 °C. The rheological tests of precursors were in situ photopolymerization (gelation time), flow measurements, and recovery behavior. The flow measurement (shear-thinning and yield stress) was carried out at a shear rate of 1–200 s^–1^ to determine the viscosity and flow behavior. For recovery behavior, the measurement was performed by using three intervals of a low shear rate (0.01 s^–1^ for 200 s), followed by a high shear rate (500 s^–1^ for 100 s) and finally, a low shear rate (0.01 s^–1^ for 200 s) to screen the viscosity recovery of precursors after extrusion. The gelation time of the precursors was quantified via in situ photopolymerization using a rheometer with an external UV lamp (BlueWave 50 UV curing spot lamp, DYMAX Corp., USA). Shear-thinning coefficients and yield stress were calculated using the Power-Law Equation and the Herschel–Bulkley model, as previously described^7,21^ and explained in the Supporting Information (eqs S-1, S-2). The viscosity of the precursors at high pH was obtained from the Cox-Merz rule (eq S-3) and transformed from the oscillatory measurement (frequency sweep, 0.1–500 rad/s, constant strain 1%).

### Prescreening of Injectability and Printability

The injectability of the hydrogels was confirmed using a commercial needle with a diameter of 22G (BD MicrolanceTM 3, Becton Dickinson S.A.). For printability, we followed simple prescreening protocols published previously:^7,21^ filament formation and stackability tests. The different precursor formulations were loaded into a 1 mL syringe and capped with 410 μm steel nozzle types. The nozzles were purchased from Nordson EFD, Germany. The precursor filament was formed in air at RT (24 °C) and at 37 °C to observe filament quality and extrudability and then deposited on the glass surface to investigate the stackability. The images of filaments were captured using a camera (Theta Lite, CMOS 1/2” USB 3.0 digital camera with fixed zoom, resolution of 1280 × 1024 pixels, Biolin Scientific, Sweden). Based on our previous studies (Figure 1), we defined the prescreening test for biomaterial ink printability and divided filaments into three categories: droplet, smooth, and irregular filament. A droplet filament indicates that the extruded precursor is too liquid and is not recommended for 3D bioprinting. A smooth filament, on the other hand, indicates that the extruded precursor exhibits smooth, uniform, and consistent filament, which is considered a good candidate for 3D bioprinting. An irregular filament indicates an over-gelation condition of the precursor, exhibiting the nonuniformed and fractured filament after being extruded from the nozzle.

### Evaluation of Printability

Filament quality checkup and 3D printing ability were assessed to determine the printability of precursor formulations. The most optimal precursor formulation was described as “ink” and printed using an extrusion-based 3D bioprinter (Brinter One, Brinter Ltd., Finland). A 410 μm steel nozzle was used in all printing tests. The ink filament checkup was done by printing lines with different pressure and printing speed values. Extrusion pressure ranged between 2000 and 3000 mbar, and the printing speed was set to 4, 6, or 8 mm/s. The filament widths were captured and measured using Image processing software (Fiji-ImageJ). The filament widths were compared to the nozzle size to determine the printability. After that, the best printing parameters were chosen to continue with multilayer printing (two and four-layered grid structure). The shapes of the pores in the printed grids were evaluated to obtain the pore geometry and *Pr* value (Figure S-11, eq S-4), as previously described.^21^ The four-layered grid structures were printed to assess the inks’ ability to support the weight of each layer while maintaining the printing resolution without collapse. The multilayered structures were postcured using the bioprinter’s integrated UV/vis LED module at a wavelength of 365 nm with 25 mW/cm^2^ intensity for 120 s.

### Hydrogel Preparation

The pH of precursor formulations was adjusted into acidic (pH 5), neutral (pH 7), and basic (pH 8). After that, the precursors were cast into the molds (2.5 mm height, diameter of 12 mm) and were left for 30 min to settle down. Next, the precursors were exposed to 365 nm UV light (25 mW/cm^2^) for 120 s (BlueWave 50 UV curing spot lamp, DYMAX Corp., USA).

### Mechanical Properties of Hydrogels

To evaluate the viscoelastic behavior of the hydrogels with and without photocrosslinking, oscillatory measurement was employed using a rheometer with a plate-to-plate geometry (12 mm of diameter). The amplitude sweep was carried out to determine the linear viscoelastic region of the materials (0.1–100% strain). Subsequently, frequency sweep measurements were carried out from 0.1 to 100 Hz at a fixed strain of 1% and at a gap distance of 2.5 mm at 25 °C. The storage and loss moduli (*G*′, *G*″) correlating to the elastic and viscous attributes of the hydrogel samples were measured and calculated into loss tangent (tan δ). Stress relaxation was also measured with a rheometer (12 mm plate-to-plate geometry) to evaluate the effect of gallic acid in hydrogels compared to plain HAMA hydrogel. The hydrogel samples were tested with 20% strain at a constant rate for 500 s, giving the stress response over time. Crosslinking density (*n*_e_, mol/m^3^) and average mesh size (ξ, nm) were estimated by calculating the difference between *G*’ and *G*″ (eqs S-5, S-6). To screen the strain recovery or self-healing behavior of the hydrogels, *G*’ and *G*″ were measured under the repeating seven cycles of low (1%) and ultrahigh oscillation strain (800%) conditions at 25 °C and oscillation frequency remained constant at 1 Hz, using 12 mm diameter stainless steel parallel plate geometry. The holding period of each cycle was set at 60 s. The self-healing properties of GA-based hydrogels were evaluated via a cutting-healing method. The hydrogels were first cut into two separate pieces, after which the cut edges were faced together at 37 °C for 30 min.

### pH-Dependent Swelling of Hydrogels

All hydrogel samples with and without postcrosslinking were immersed in 0.1 M phosphate-buffered saline (PBS) solution with different pH (5, 7, and 9) to examine their stability (K_2_HPO_4_ and KH_2_PO_4_ were varied from 5.841 and 94.16 mM to 93.48 and 6.523 mM to obtain the desired pH). The hydrogels were maintained at 37 °C ± 0.5 °C in a shaking incubator at 90 rpm until various time points (0, 1, 2, 3, 5, 7, and 15 days). At the zero time point, the samples were defined with a weight of *W*_0_. At every time point, the samples were removed from the solution, and the residual solution from the surface was removed to obtain the *W*_s_. The swelling ratio was calculated as *W*_s_/*W*_0_.

### Degradation Study

Enzymatic degradation of the material was performed using hyaluronidase at a concentration of 50 U/mL in DPBS at pH 7.4. Three parallel hydrogel samples of 250 μL HAMA15, HAGA10-HAMA15, and HAGA20-HAMA15 were prepared in the molds. Similarly to the swelling test, hydrogel samples were submerged in 1 mL hyaluronidase DPBS solution until various time points (0, 1, 2, 3, 5, and 7 days). At the zero-time point, the samples were defined as having an initial weight of *W*_0_. At every time point, the samples were removed from the hyaluronidase buffer, the residual buffer from the surface was removed to obtain the *W*_m_, and the enzyme buffer was replaced after each measurement. The degradation weight percentage was calculated as *W*_m_/*W*_0_ × 100.

### Adhesive Properties

A tack test was performed for HAMA and HAGA-HAMA using a rotational rheometer at RT to observe the adhesive properties. The protocol has been reported in a previous study.^2^ In brief, chicken skins and porcine muscles (freshly purchased from the market) were carefully cut into circular sheets having a 12 mm diameter and attached to the upper and bottom plates. Next, the precursors were injected between two tissue layers. The upper plate with the attached animal tissue was then pressed with a uniform compressive force (0.1 N) for 120 s to settle the tissue and the precursor. Subsequently, hydrogels were formed by in situ photocrosslinking with a UV lamp for 120 s. Thereafter, the upper plate was pulled up in axial motion at a constant velocity of 20 μm/s. The change in axial force was recorded at the point of detachment. A graph was then plotted to observe the influence of gallic acid on the adhesive properties of the precursor compared to HAMA without gallic acid. Each test contained five parallel samples. The tissue used for the adhesive study was moist throughout the measurement.

### Antioxidant Properties

Free radical scavenging activity of HAGA-HAMA was evaluated using the DPPH (2,2,1-diphenyl-1-picrylhydrazyl) method.^36^ HAGA was dissolved in DI water at 30 μg/1 mL concentration, followed by 1 mL of DPPH solution (1 mg/12 mL in methanol). After incubation at 25 °C for 30 min, the absorbance was measured at 517 nm using a UV–vis spectrophotometer. The DPPH scavenging activity (%) was calculated from eq 1.
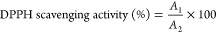
1where *A*_1_ is the absorbance of DPPH solution in the presence of samples, and *A*_2_ is the absorbance of blank DPPH solution that was used under the same reaction conditions in the absence of synthesized polymers.

### Statistical Analysis

The results of the oscillatory measurements were presented as mean ± standard deviation (SD). The analysis was performed using Student’s *t*-test to determine the differences between groups, and the significance was defined at *p* < 0.05.

## Results

### Development of pH-Responsive Precursors

The HAGA precursors were synthesized with calculated modification degrees of 10 and 20% and were obtained as ∼12 and ∼21%, as confirmed by H1-NMR (Figure S-8A,B and Figure S-9A,B). The degree of methacrylation of the HAMA15 precursor was approximately 16%, quantified by H1-NMR (Figure S-10A,B). According to the appearance, the precursors were liquid under acidic conditions (pH 3–5), gained more viscosity at pH 7.5–8, and became true hydrogels under basic conditions (pH 8.5–9). The preliminary testing was performed to screen the precursors’ injectability and printability after extruding from the nozzle. In Figure S-12, at pH 3–7.5, the precursor displayed a droplet-like filament, whereas the filaments of the precursor at pH 7.5–8 were coherent. The precursor at pH > 8 was unable to form a coherent filament; instead, it was irregular and too solid. In addition, 5% w/v of HAMA precursor displayed a liquid-like filament and was not a good candidate for 3D bioprinting (Figure S-15A).

### Flow Behavior of pH-Responsive Precursors

**Figure 3:**
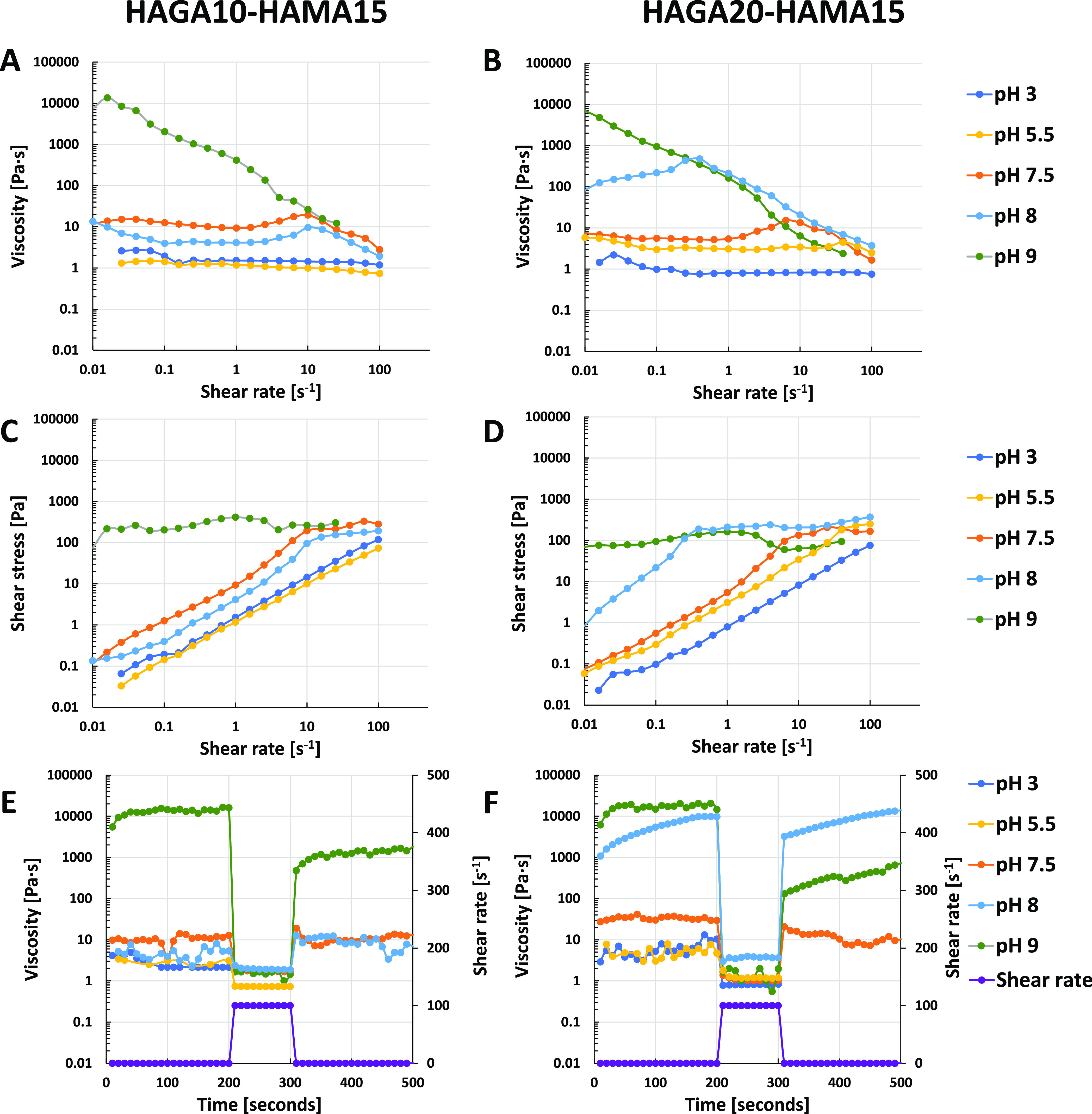
Rheological measurements of precursor mixtures of HAGA10-HAMA15 and HAGA20-HAMA15 at different pH (3, 5.5, 7.5, 8 and 9): shear-thinning (A, B), yield stress (C, D), and recovery behavior (E, F) at 37 °C.

Figure 3 illustrates the flow curve of shear-thinning and recovery behavior of precursors at different pH. The values of shear-thinning coefficients and yield stress were used to explain the injectability and printability. In detail, all precursors at low pH (3–5) exhibited low viscosity and behaved liquid-like. This was confirmed by the shear-thinning coefficients of *n* > 0.9 (Table S-1), describing the precursor as a Newtonian fluid. The precursors started to gelate when the pH reached 7.5, resulting in higher viscosity as the pH increased.

At acidic and neutral pH, all precursor formulations were liquid and partially slipped out of the plate-to-plate geometry after applying the shear. According to the viscosity curve of HAGA10-HAMA15 at pH 7.5–8 (Figure 3A), Newtonian behavior was observed at a low shear rate, revealing the extended plateau region, finally demonstrating non-Newtonian behavior at 10 s^–1^ shear rate. The values of shear-thinning coefficients are listed in Table S-1. On the other hand, as shown in Figure 3B, HAGA20-HAMA15 at pH 5–7.5 was weakly shear-thinning as the viscosity started to drop at a high shear rate (above 10 s^–1^). HAGA20-HAMA15 at pH 8 showed improvement in shear-thinning behavior at a shear rate above 1 s^–1^, giving *n* < 0.2 (Table S-1). In addition, HAGA10-HAMA15 and HAGA20-HAMA15 at pH 8 did not have high yield stress, implying that high printing pressure is not required to extrude the material (Figure 3C,D). The recovery behavior graphs are represented in Figure 3E,F. HAGA20-HAMA15 at pH 8 rapidly recovered to its original viscosity (∼80%) after removing the high shear rate. In contrast, at pH 9, HAGA10-HAMA15 and HAGA20-HAMA15 lost half of their viscosity after removing the shear.

At pH 9, both HAGA10-HAMA15 and HAGA20-HAMA15 became true hydrogels. The Power-Law model was applied, and shear-thinning coefficients of *n* < 0.2 were obtained. In contrast, they required high shear stress to reduce the viscosity and could not recover their original viscosities (∼40–50% recovery). Based on the rheological results, HAGA20-HAMA15 at pH 8 provided high viscosity, proper yield stress, shear-thinning properties, and recovery behavior. Hence, it was selected as the biomaterial ink candidate for injecting and 3D printing tests. In addition, in situ photopolymerization (Figure S-13) shows the gelation time of all precursor formulations at different pH after being exposed to UV light (storage modulus as a function of time).

### Evaluation of Printability

**Figure 4:**
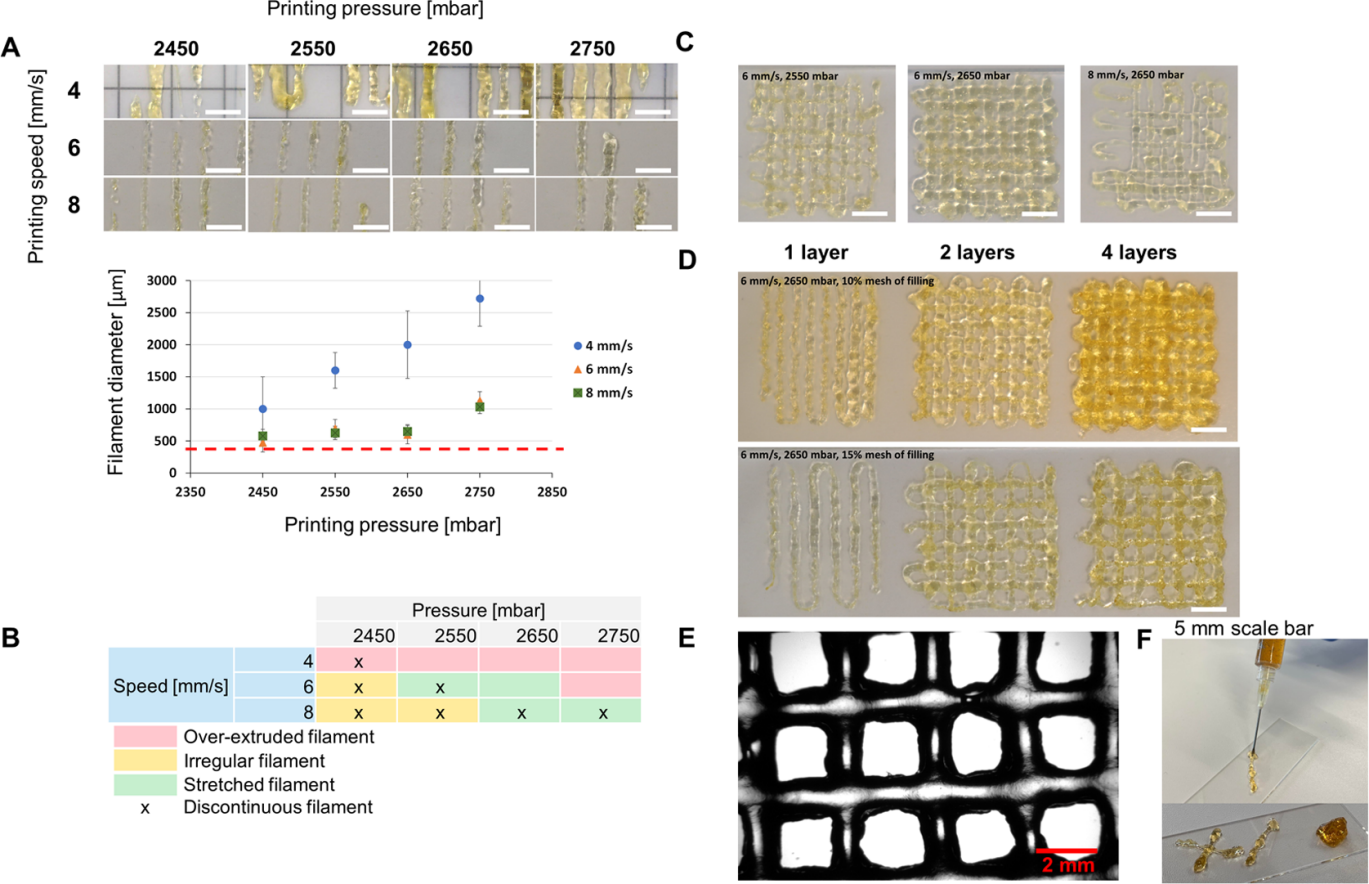
Printability of biomaterial inks (HAGA20-HAMA15 at pH 7.5–8, 37 °C) and 3D printing tests. (A) Filament of printed biomaterial inks with various pressure and printing speed values. The graph illustrates how filament diameter is affected by pressure and printing speed. The red line is used as a guideline to compare the filament diameter with the actual nozzle size. The error bars indicate the standard deviation of filament diameter for each ink, presented as mean (*n* = 10) ± SD. (B) Printability window: an over-extruded filament (red color), irregular filament (yellow color), stretched filament (green color), or discontinuous filament (x symbol). (C) Images of two-layer printed grids to screen the optimal printing parameters. (D) Images of multilayer printing of one, two, and four layers using the optimal printing parameters and different filling percentages to determine the achievable printing resolution. (E) Example of a microscopic image of an optimal printed grid structure for *Pr* value calculation and stackability of 2 filament layers (6 mm/s, 2650 mbar). (F) Example of the prescreening results of injectability and stackability of biomaterial inks.

After the printability of precursors was prescreened using filament analysis (filament formation and stackability) and rheology (shear-thinning, yield stress, and recovery behavior), the integrity of multilayered constructs and the effect of printing parameters were evaluated using *Pr* value calculation and filament width measurement. Figure 4A shows the printed filaments at different printing conditions. Low printing speed (4 mm/s) fed excessive ink on the printing bed, resulting in over-deposited filaments. However, the ink started to extrude at the pressure of 2450 mbar and could not form a smooth filament. The printing speeds of 6 and 8 mm/s resulted in continuous thin filaments having thickness close to the nozzle diameter. Figure 4A also shows the measured filament thicknesses compared to the nozzle size. However, some discontinuous filaments were observed at the pressure values of 2450 and 2550 mbar. The thickness of filaments can be used to predict the accuracy of the printing process.^37^ The thickness of continuous filaments was around 577 ± 8 μm. The printing pressure of 2650 mbar and a speed of 6 mm/s resulted in a continuous filament and were chosen for multilayered grid (2 × 2 cm^2^) printing. The filament characteristics and printing parameters were collected in the table and marked as the printability window to narrow the printing parameters (Figure 4B). The red color indicates over-extruded filaments caused by too high printing pressure and speed. The irregularly shaped filaments caused by unfeasible printing speed with proper pressure were marked with yellow. The green color shows the part of the printability window where the printing pressure and speed meet the minimum requirements, giving well-defined filaments. The x symbol demonstrates the filament breakage during the printing, yielding poor printing results. Figure 4C visualizes how irregular grid structures were formed if inappropriate printing parameters were chosen. Figure 4D shows the successful 3D printed grids (2 and 4 layers) using optimized printing parameters with different degrees of filling for the CAD model (to change the size of the internal pore size in the grid). The printability of HAGA20-HAMA15 was evaluated by varying the printing parameters, such as printing pressure (2450–2750 mbar) and speed (4–8 mm/s). Ideally, the proper printing parameters provide stability and shape fidelity for the printed structure, which allows the 3D stacking of filaments in a layer-by-layer fashion. Figure 4F shows the result of injectability and stackability of HAGA20-HAMA15 using a commercially available needle at RT and 37 °C. The material was stackable on the glass slide. Subsequently, the *Pr* values were calculated from the grid constructs from the optimal printing parameters (Figure S-16 and Table S-2).

### Viscoelastic Properties of Hydrogels

**Figure 5:**
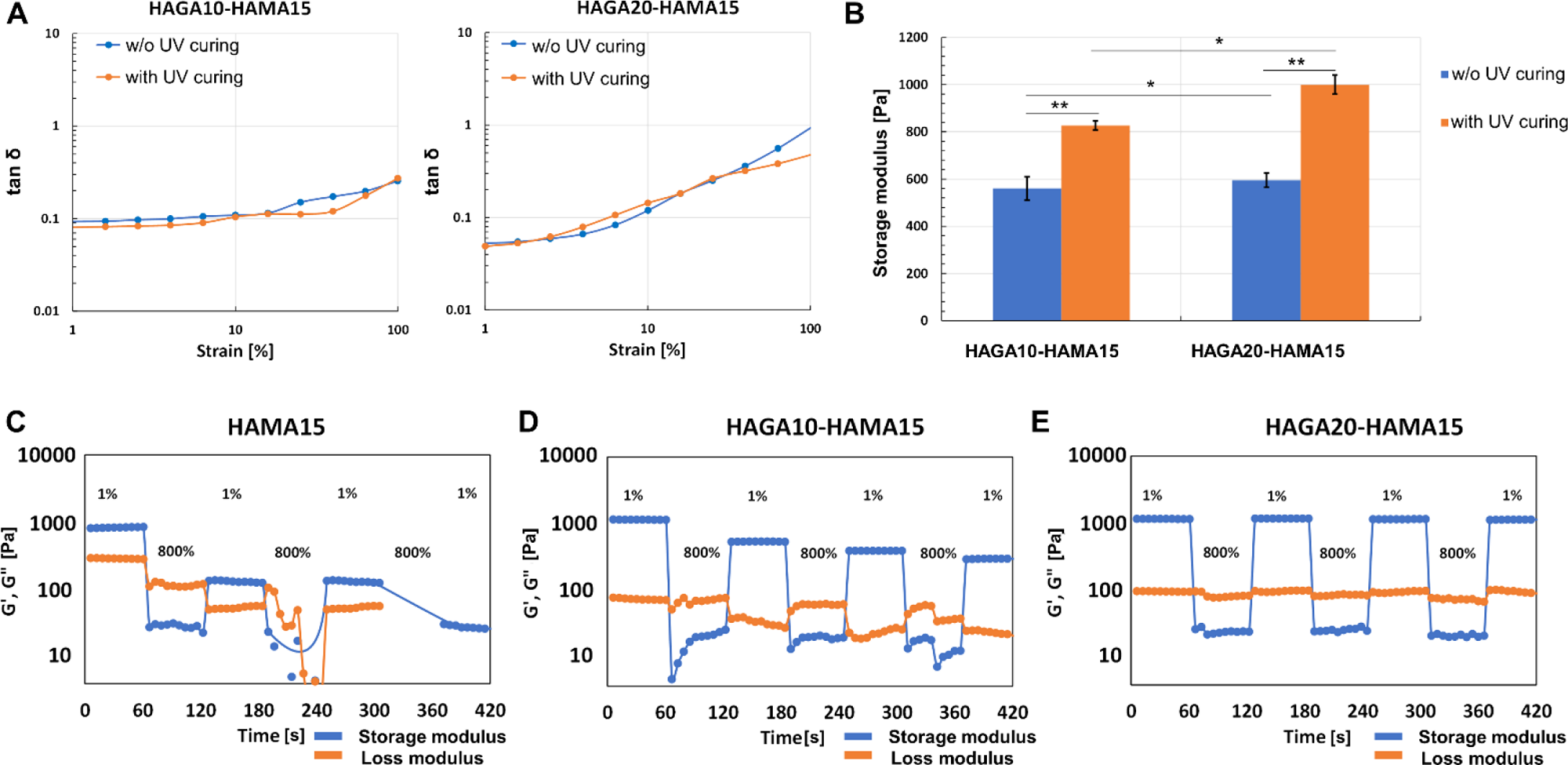
Oscillatory measurements of all hydrogel samples, tested using frequency and amplitude sweeps. (A) tan δ value, calculated from the ratio between *G*’ and *G*″ from the amplitude sweep to observe the viscoelasticity of hydrogels (with and without UV). (B) Storage moduli of hydrogels (with and without UV) obtained from the linear region of amplitude and frequency curves. The error bars indicate the standard deviation of storage modulus for each ink, presented as mean ± SD (*n* = 10, *p < 0.05, **insignificant). (C–E) Comparison of strain recovery behavior of hydrogels with the complementary network (HAGA10-HAMA15 and HAGA20-HAMA15) and without the complementary network (HAMA15). The strain recovery behavior was measured through seven cycles of strain (1% strain → 800% strain → 1% strain).

**Figure 6:**
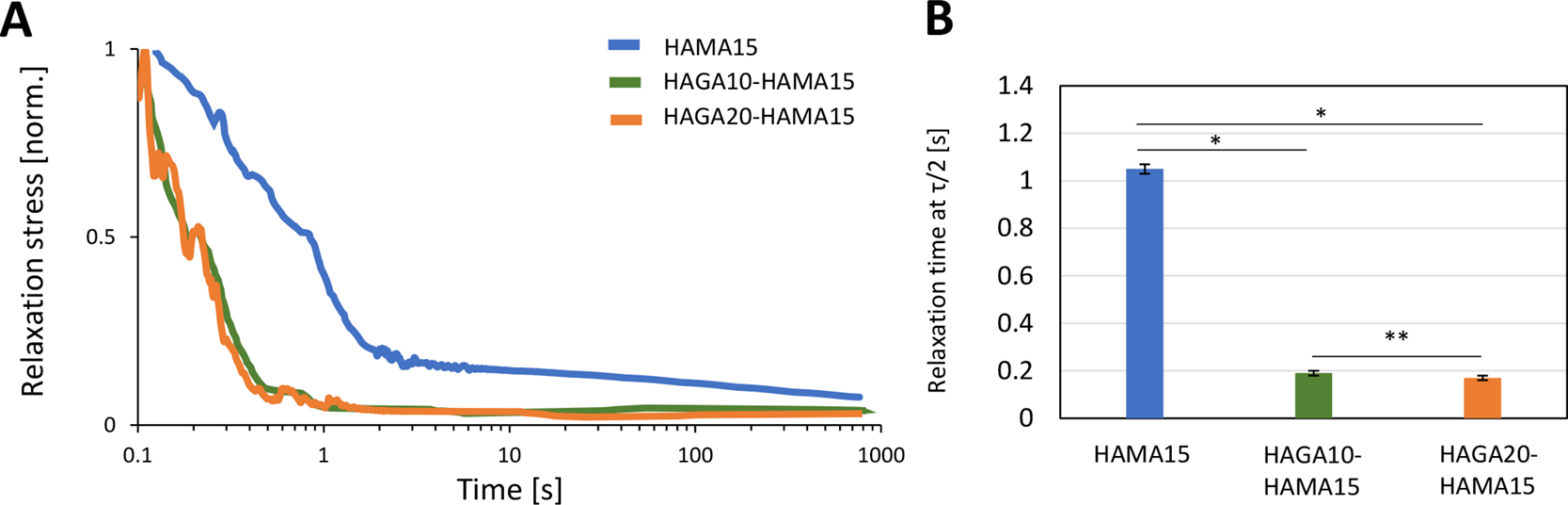
Stress relaxation tests on HAGA-HAMA and HAMA hydrogels. (A) Hydrogel samples were tested with 5% strain, which was then held at a constant rate for 1000 s. (B) Quantification of stress relaxation in the time scale at which the stress is relaxed to half of its initial value. The error bars indicate the standard deviation of storage modulus for each ink, presented as mean ± SD (*n* = 10, *p < 0.05, **insignificant).

The storage moduli of hydrogels were evaluated using oscillatory measurement (amplitude and frequency sweeps) with and without photocrosslinking (Figure 5). As mentioned in the Materials and Methods section, HAGA-HAMA formulations were adjusted to basic pH to be gelated into true hydrogels. The rheological analyses represented that with and without UV curing, gels remained stable during the rheological testing. Both formulations consistently yielded higher storage moduli after photocrosslinking (560 ± 11, 827 ± 26, 595 ± 12, and 1060 ± 25 Pa for HAGA10-HAMA15 without UV-curing, UV-cured HAGA10-HAMA15, HAGA20-HAMA15 without UV-curing, and UV-cured HAGA20-HAMA15, respectively). Moreover, Figure 5A demonstrates that UV-cured and hydrogels without UV-curing resulted in viscoelasticity and high stability (tan δ values lower than 1), but there were no significant differences between the two formulations (Figure 5B). The average mesh sizes (ξ) and crosslinking densities (*n*_e_) were calculated using eqs S-5 and S-6 and are listed in Table S-3. Stress relaxation on HAMA and HAGA-HAMA gels was evaluated to observe the effect of GA functionalization in the hydrogel networks. As shown in Figure 5C–E, the dynamic strain recovery properties of the complementary network hydrogels were assessed using the continuous seven-step strain (1% strain → 800% strain → 1% strain). At high strain (800%), the hydrogels reached the critical strain, which was converted into a viscous state (*G*″ dominates *G*’). At low strain (1%), the hydrogels exhibited an elastic state (*G*’ dominates *G*″). During the cyclic test, the rapid transition between elastic and viscous states between low and high strain indicates the strain recovery behavior of the hydrogels. The HAGA-HAMA hydrogels showed high elastic recoverability of the polymeric networks (especially HAGA20-HAMA15), suggesting the dynamic nature of the complementary network between gallol moieties and photocrosslinking. In contrast, HAMA15 groups lost their original properties after the first high strain. Figure 6A confirms that HAGA-HAMA hydrogels have enhanced stress relaxation behavior. When comparing the relaxation behavior of HAMA15, HAGA10-HAMA15, and HAGA20-HAMA15, we observed that the HAGA-HAMA groups displayed a faster relaxation time (at 0.5 relaxation stress), which was approximately 0.2 ± 0.01, 0.18 ± 0.01, and 1.05 ± 0.02 s for HAGA20-HAMA15, HAGA10-HAMA15, and HAMA15 respectively, as shown in Figure 6B. However, the relaxation amplitude and relaxation time between HAGA10-HAMA15 and HAGA20-HAMA15 were not significantly different.

### pH-Dependent Swelling of Hydrogels

**Figure 7:**
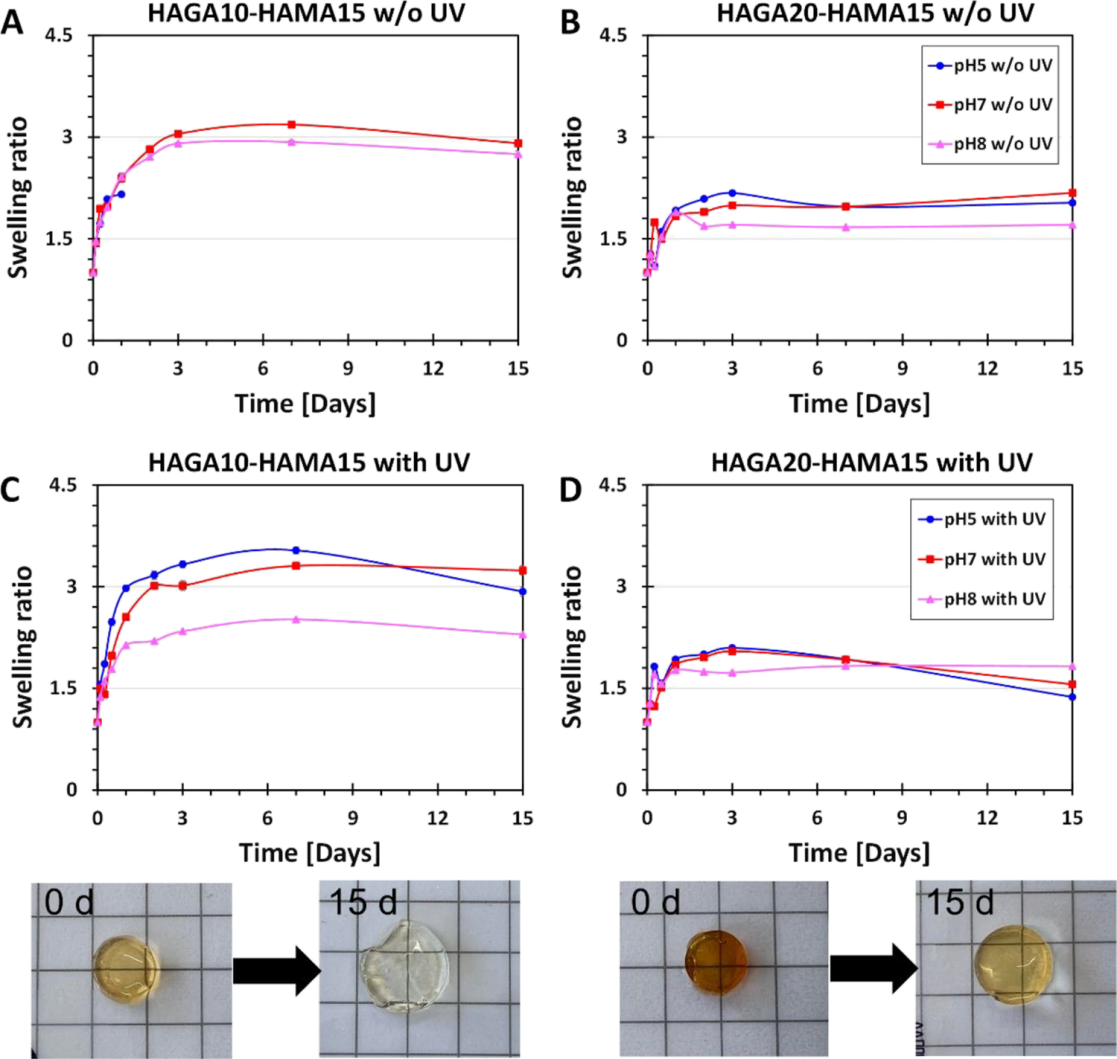
Time-dependent swelling behavior of HAGA10-HAMA15 and HAGA20-HAMA gels (*n* = 10, the error bars indicate the SD) as a response to different pH (5–8). The swelling ratio of hydrogels (A) HAGA10-HAMA15 and (B) HAGA20-HAMA15. The hydrogels before and after swelling are shown here as examples, 1 cm^2^ grid scale.

The swelling studies were performed to investigate the stability of the HAGA-HAMA gels (with and without UV curing) under physiological conditions (pH 7.4) and at acidic conditions (pH 5.0), in which the GA modification starts to degrade, and at basic conditions (pH 8.0) (Figure 7). HAGA10-HAMA15 hydrogels without photocrosslinking (Figure 7A) disintegrated after the first time point of observation in the acidic conditions, but HAGA20-HAMA15 hydrogels without photocrosslinking (Figure 7B) were stable until the end of the observation. In contrast, both UV-cured HAGA10-HAMA15 (Figure 7C) and HAGA20-HAMA15 (Figure 7D) hydrogels showed rapid initial swelling followed by degradation in acidic buffer, but the HAGA20-HAMA15 gels displayed a significantly lower swelling ratio (∼4.1 ± 0.03 and ∼2.2 ± 0.02 swelling ratio, respectively). Under physiological conditions and at basic pH, HAGA20-HAMA15 gels (with and without UV) were stable, but HAGA10-HAMA15 gels at pH 7 swelled more than HAGA20-HAMA15 gels.

### Adhesive Properties

**Figure 8:**
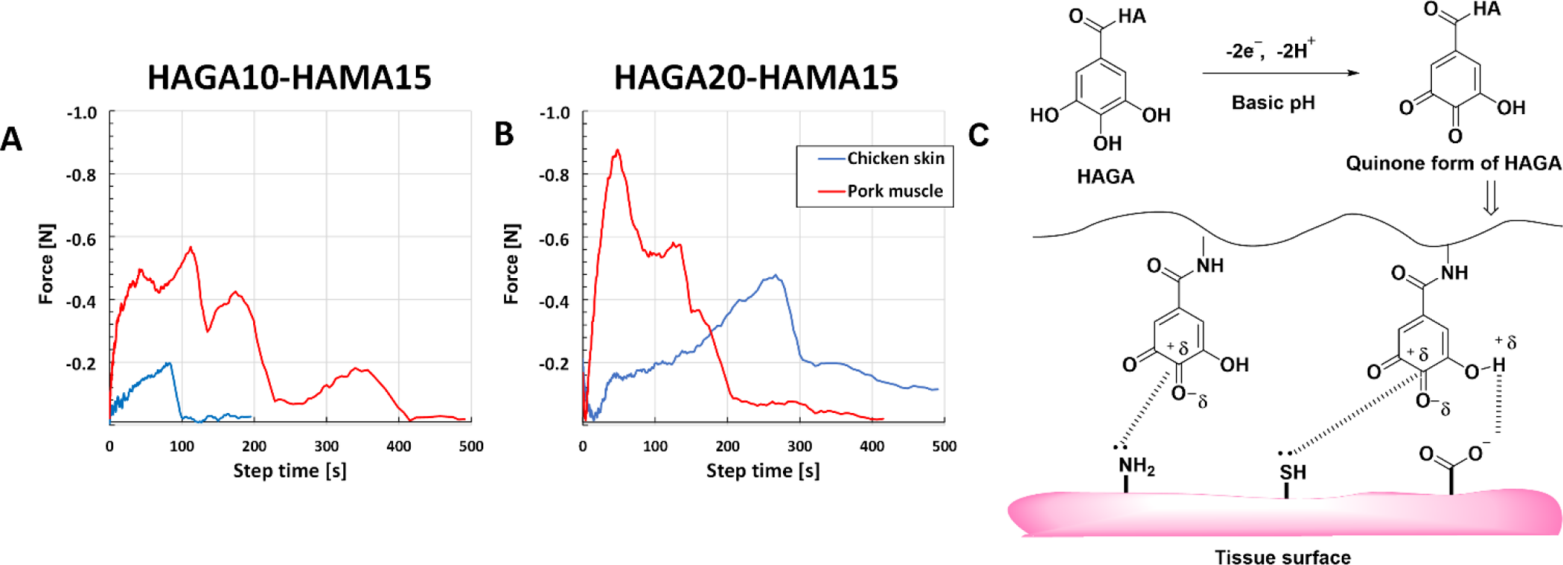
Measurement of tissue adhesion force of hydrogels (*n* = 4) (A) HAGA10-HAMA15 and (B) HAGA20-HAMA15 by the tack adhesion test. The precursors were in situ photocrosslinked and adhered to the surface of animal tissues. (C) Adhesion chemistry between hydrogels and the tissue surface due to nucleophilic group interactions and quinone.

A tack test was performed to investigate the tissue adhesive properties of different inks using chicken skin and porcine muscle. Both HAGA10-HAMA15 (Figure 8A) and HAGA10-HAMA15 (Figure 8B) showed tissue adhesive properties. However, a higher modification degree of GA (HAGA20) required higher force to pull the tissue from the in situ photocrosslinked hydrogels (negative force) compared to gels with a lower modification degree of GA (HAGA10). Figure 8C shows the mechanism of wet adhesion on tissue surfaces.

### Antioxidant Properties

**Figure 9:**
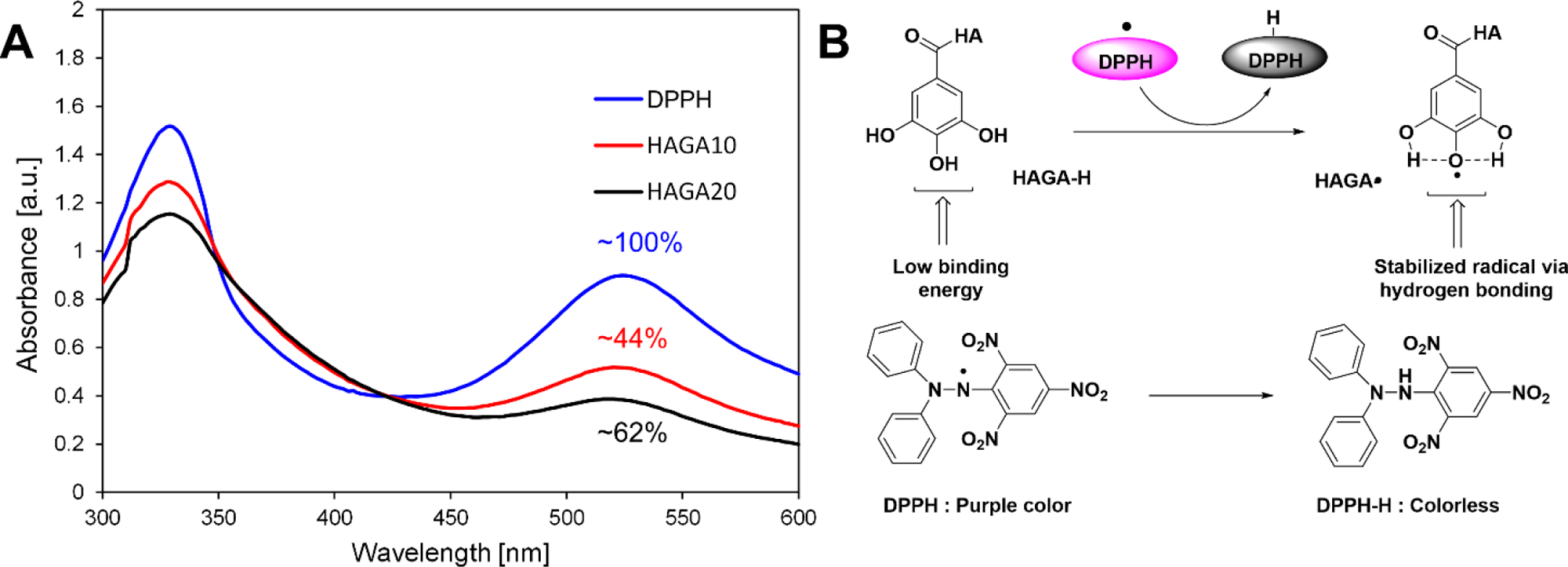
Antioxidant properties of HAGA10 and HAGA20. (A) UV–vis spectrum of HAGA10 and HAGA20 exhibited a reduction of absorbance at 530 nm, compared to DPPH alone. (B) DPPH radical scavenging mechanism that is responsible for the antioxidant activity of HAGA.

A DPPH radical scavenging assay was used as a preliminary assessment of the changes in the antioxidant properties upon modification of HA with GA. The DPPH reagent underwent a visual change in color from deep purple to deep orange in HAGA10 and HAGA20 due to the antioxidant properties imparted by GA. The antioxidant properties of GA were confirmed again by comparing it with pure DPPH, which did not change its color, and was considered as 100% absorption. The UV–vis spectroscopy measurement of 30 μg/mL HAGA10 and HAGA20 in the presence of DPPH displayed ∼44 and ∼62% reduction in absorption (Figure 9A), indicating potential antioxidant properties. Figure 9B illustrates the mechanism of radical quenching by gallol moieties.

## Discussion

Among all kinds of stimuli-responsive hydrogels, pH-responsive hydrogels have been extensively explored due to their potential for various applications, such as injectable and self-healing hydrogels as well as drug delivery systems.^31^ In addition, the pH-responsiveness in precursors enables control over the precursor viscosity and mechanical, swelling, and degradation properties of hydrogels. The pH-responsive precursors can be bioprinted at neural-basic pH (7.3–9.8) on damaged skin^31^ and be triggered to degrade at low pH (4–6) on healed skin.^32^ In bioprinted wound dressing applications, the pH-responsive non-Newtonian precursors enable the in situ bioprinting of the precursor in the wound and allow the shape-specific fitting and stability of the printed construct.^38^

In this study, we synthesized pH-responsive hyaluronic acid-(HA)-based precursors grafted with the gallic acid (GA) moiety (the degrees of GA functionalization were ∼10 and 20% with respect to the disaccharide repeat units). GA was conjugated to polysaccharides via carbohydrazide linkages utilizing the carboxylic group on the polymer backbone and hydrazide group from amine-functionalized GA using carbodiimide coupling chemistry.^1,39^ The carboxylate residues of GA were modified to a hydrazide derivative as they are known to undergo proficient EDC coupling at acidic pH (4.7–4.8). The successful conjugation of GA was confirmed as the solution turned light brown at basic pH (∼8), indicating that GA functionalization was successful in the HA backbone, as also demonstrated in our previous study of GelMAGA.^36^ We hypothesized that the precursor blend (HAGA-HAMA) would provide the pH-responsive properties necessary to improve both printability and injectability. We decided to test higher (20%) and lower (10%) gallic acid modifications to understand the effect of the gallic moiety on pH responsiveness. To simplify the study, the ratio between HAMA and HAGA was fixed to 1:1, and the HAMA hydrogel was used as a control.

A series of rheological characterizations were performed to study the pH-dependent precursor properties. Shear-thinning behavior in non-Newtonian precursors has been used in several research studies to show the printability of precursors.^20,40^ The Power-Law model was applied to the flow curve to calculate the shear-thinning coefficients (*n* and *K* values). These coefficients were used to prescreen whether the precursor is injectable or printable. In general, the linear region from the viscosity–shear rate curve has been used for the Power-Law trendline fit.^7,18,36^ HAGA-HAMA precursors undergo a rapid sol–gel transition between pH 7.5 and 8, which results in increased viscosity at higher pH levels due to denser crosslinked network formation via oxidized gallol moieties. However, the precursor at basic pH became the true hydrogel and slipped from the parallel plate at high-velocity centrifugal movement during the measurement. Therefore, the Cox–Merz rule was applied to convert the frequency sweep to a viscosity-shear rate graph (eq S-3 and Figure S-14). The Cox–Merz rule is a correlational relationship that can predict the shear rate-dependent viscosity based on the oscillatory data.^41^ We applied the rule when the measurement of precursors became impossible at a shear rate above 5 s^–1^ due to the rotational movement of the geometry. Precursors at acidic pH had an *n* value close to 1, which led to droplet formation. On the other hand, precursors at neutral and basic pH had an *n* value below 0.2, making them highly shear-thinning. To further evaluate the printability, the recovery behavior of the precursors was evaluated. Upon removal of the high shear rate, HAGA20-HAMA15 at pH 8 recovered its initial viscosity, suggesting the thixotropic behavior.

In general, the precursors for injecting and 3D bioprinting must exhibit shear-thinning behavior, displaying a decreasing dynamic viscosity as a function of increasing shear rate and also have the recovery behavior as well as the ability to stack layer-by-layer during printing.^18,38^ According to the results, precursor formulations at neutral pH could not provide enough stackability, as the filaments merged after being deposited on the substrate. We have recently shown that the rheological data alone cannot guarantee a 100% success rate for printing.^7,21^ To confirm the printability, the printability window was formulated to provide the influence of printing pressure and printing speed on the filament diameter and quality, giving the proper printability data. When the pressure was increased, filaments swelled, leading to poor accuracy. Increasing the printing speed resulted in thinner filaments but could also cause discontinuous filaments if the speed did not match the pressure. To investigate the feasibility of 3D printing HAGA20-HAMA15 at pH 7.5–8, 3D cylinders were printed to observe the stackability of multilayered structures (Figure S-17). We found that the cylinders with lower heights of 1 and 2.5 mm could stack successfully. However, the structural integrity of 5 mm cylinders was poor due to the ink’s inability to support its weight, which resulted in structural collapse after the fifth layer was printed.

The pH-induced crosslinking, together with photocrosslinking, provided a complementary network in the HAGA-HAMA hydrogel. To verify the gallol-mediated complementary network formation in the HAGA-HAMA hydrogels, we performed oscillatory and stress relaxation measurements. We observed the complementary network of HAGA-HAMA before and after photocrosslinking, and we found that HAGA-HAMA displayed more stress relaxation than HAMA alone. According to Chaudhuri et al., living tissues behave viscoelastic and have stress relaxation.^42^ The addition of photocrosslinking led to increased storage modulus, higher crosslinking density and more elastic gels (tan δ), indicating more stable matrix formation. In general, GA functionalized hydrogels possess strain recovery and self-healing behavior.^1,2,34^ Therefore, a series of rheological recovery tests were conducted with *G*’ and *G*″ under the seven cycles of low and high strain to determine the superiority of complementary networks in hydrogels. After the first cycle, HAMA hydrogels lost their initial *G*’ value because HAMA hydrogels were covalently formed by a single network, resulting in brittleness in the hydrogel structure.^43^ The strain recovery of hydrogels may increase due to the addition of secondary crosslinking, such as interpenetrating and complementary network.^1,13,44,45^ According to the results, the *G*’ of the HAGA10-HAMA15 and HAGA20-HAMA15 hydrogels under high dynamic strain decreased due to the deformation of the hydrogel network. After the low strain, they quickly returned to the original *G*’ value as the hydrogel construct recovered, especially in hydrogels with higher GA modification. In addition, the self-healing properties of the HAGA20-HAMA15 hydrogels were evaluated using a cutting-healing test. After 30 min of incubation at 37 °C, the separated hydrogel discs were merged with each other (Figure S-18). However, HAGA-HAMA hydrogels at high basic pH levels produced a brownish color, which might reduce the transparency of the precursor, affecting the UV light penetration and hindering photocrosslinking. Hence, the results of in-situ photorheology (Figure S-13) showed a slight improvement in storage modulus upon UV exposure. Moreover, GA has been proven to be an antioxidant, leading to radical inhibition, ultimately reducing the degree of photocrosslinking.

The pH-dependent swelling was investigated to further confirm the concept of the complementary network and controlled swelling properties of HAGA-HAMA hydrogels. The acidic media pH led to higher water uptake and increased the swelling of hydrogels. At basic and neutral media pH, the GA hydrogels exhibited stable swelling over the period of observation. The HAGA20-HAMA15 hydrogels illustrated a slower swelling rate than the HAGA10-HAMA15 hydrogels, especially after day 1. The complementary network limited the hydrogel swelling and reduced the average mesh size (ξ), resulting in reduced water uptake into the hydrogels. According to the previously published studies,^46,47^ crosslinking density (*n*_e_) and average mesh size (ξ) influence hydrogel’s swelling capacity. The higher crosslinking density results in additional network formation; subsequently, the network structure of hydrogel is formed, which reduces the water absorption. To further confirm the gallol-mediated complementary network formation in the HAGA-HAMA hydrogels, an enzymatic degradation study was performed in the presence of hyaluronidase in DPBS at pH 7.4, as shown in Figure S-19. Both groups of HAGA-HAMA hydrogels exhibited slower degradation than the plain HAMA hydrogel, especially after day 1. The HAMA hydrogels degraded quickly after 4 days, and the remaining mass was lost at the end of the observation. The interpenetrating crosslinking between photocrosslinking and gallol-mediated network in the HAGA-HAMA hydrogels limited their enzymatic degradation by bulk erosion, resulting in a slower degradation that proceeded through surface erosion.

In general, HA has been shown to enhance wound healing and modulate inflammation.^34^ GA possesses a large variety of bioactive characteristics, including anti-carcinogenic, anti-mutagenic, and anti-inflammatory properties.^34^ Recently, we have shown that a GA-functionalized GelMA displayed antioxidant and tissue adhesive properties.^36^ We aimed to incorporate the advantages of two moieties into our precursor by grafting GA on the HA backbone. The tissue adhesive behavior of the HAGA-HAMA was determined by a tack test. HAGA20-HAMA15 displayed a high negative force with chicken skin and pork muscles compared to HAGA10-HAMA15. Our developed HAGA-HAMA blends showed a significantly stronger adhesion compared to previous reports on tissue adhesive hydrogels with similar functionalization.^2^ The surface of biological tissues has a variety of amino acids that has several nucleophilic groups available for interaction with electrophilic groups.^48^ Although the exact mechanism for tissue adhesion is unclear, we anticipate that catechol groups on the gallic acid oxidized to a quinone provide adhesion to biological tissues by forming covalent bonds with the residual nucleophilic moieties (amines, thiol, and hydroxyl groups) on the tissue surfaces.^49^ This tissue adhesion in a moist environment paves the path for developing the class of biomaterials that can be used as bio-glue in contact with the body fluids. The biomaterial inks without tissue adhesion may detach from the substrate or the formerly printed layers during the printing process, which results in poor resolution and structural deformation.

Antioxidant properties of HAGA-HAMA were confirmed by the DPPH radical quenching assay, which was used as a preliminary test to estimate the antioxidant properties upon the incorporation of gallic acid. The catechol group is known to scavenge free radicals and show antioxidant properties. In addition, GA derivatives have also been found in many phytomedicines with various biological and pharmaceutical activities, including free radical scavenging effect, induction of cancer cell apoptosis, and protection of cells from UV- or irradiation-induced damage.^49^ As shown in Figure 9B, the GA derivatives demonstrate antioxidant properties due to the formation of radical intermediate on the para-hydroxyl group stabilized by strong intramolecular hydrogen bonding.^50^

## Conclusions

The HAGA precursor with pH-responsive properties can be blended with other nonviscous precursors to improve the processability (printability, type of crosslinking and gelation time), printing accuracy, and tissue attachment. The complementary network of HAGA-HAMA hydrogels formed via pH change and photocrosslinking enhanced the viscoelasticity properties and the stability of hydrogels. HAGA-HAMA hydrogels exhibited controlled swelling properties, were capable of swelling under acidic conditions and became stable at neutral and basic pH. Moreover, the presence of GA moiety in the hydrogel network offered antioxidant and tissue-adhesive properties. Overall, we provided the fundamental connection of chemistry, rheology, and 3D fabrication, which can help to standardize the 3D bioprinting protocol from bioink development to post-processing. Moreover, our pH-responsive precursor is capable of opening a new venue for 4D bioprinting with several applications in tissue engineering and drug delivery.

## Supporting Information Available

Synthesis of hyaluronic acid methacrylate (HAMA), synthesis of a hydrazide derivative of gallic acid (GA-Hyd), synthesis of gallic acid conjugated hyaluronic acid (HAGA), ^1^H-NMR spectra of methyl ester, 13C-NMR spectra of gallic acid methyl ester, ^1^H-NMR spectra of GA-Hyd, 13C-NMR spectra of GA-Hyd, ^1^H-NMR of HAGA10, ^1^H-NMR of HAGA10 (expanded), ^1^H-NMR of HAGA20, ^1^H-NMR of HAGA20 (expanded), ^1^H-NMR of HAMA15, ^1^H-NMR of HAMA15 (expanded), pore geometry evaluation and calculation of the printability (*Pr*) value, filament quality of plain HAMA15, HAGA10-HAMA15 and HAGA20-HAMA15 at pH 3–5, 7.5–8 and >8, gelation time of HAGA10-HAMA15 and HAGA20-HAMA15, Power-Law model equation, Cox-Merz rule explanation, HAGA10-HAMA15 and HAGA20-HAMA15 after applying Power-Law and Cox-Merz rule, values of shear-thinning coefficients, HAMA15 and HAGA20-HAMA15 (neutral pH) printing test, *Pr* value calculation (equation), the example of biomaterial ink printability assessment from the macrostructure (HAGA20-HAMA15 at pH 7.5–8, *Pr* values of printed grid structures, 3D printed cylinder structures of HAGA20-HAMA15 at pH 7.5–8, crosslinking density and mesh size calculation equation, the calculated mesh size and crosslinking density values, self-healing behavior, and degradation study (PDF)

## Abbreviations

HA: hyaluronic acid
GA: gallic acid
HAGA: gallic acid-functionalized hyaluronic acid
HAMA: hyaluronic acid methacrylate
PI: photoinitiator
