# Supplementary material for: pH-Responsive Gallol-Functionalized Hyaluronic Acid-Based Tissue Adhesive Hydrogels for Injection and Three-Dimensional Bioprinting
Source: ACS Appl Mater Interfaces. 2023 Jul 6;15(28):33972–84. doi: 10.1021/acsami.3c02961 (PMC10360037; doi:10.1021/acsami.3c02961)
Supplement: Supplementary file 1 — am3c02961_si_001.pdf [file am3c02961_si_001.pdf]

pH-Responsive Gallol Functionalized Hyaluronic Acid-based Tissue Adhesive Hydrogels for Injecting and 3D Bioprinting

Hatai Jongprasitkul[a], Vijay Singh Parihar [a,], Sanna Turunen [a,b], Minna Kellomäki[a]*

[a]Biomaterials and Tissue Engineering Group, BioMediTech, Faculty of Medicine and Health Technology, Tampere University, 33720 Tampere, Finland

[b]Brinter Ltd, 20520 Turku, Finland

Corresponding Author

*Vijay Singh Parihar (vijay.parihar@tuni.fi)

1. Synthesis of hyaluronic acid methacrylate

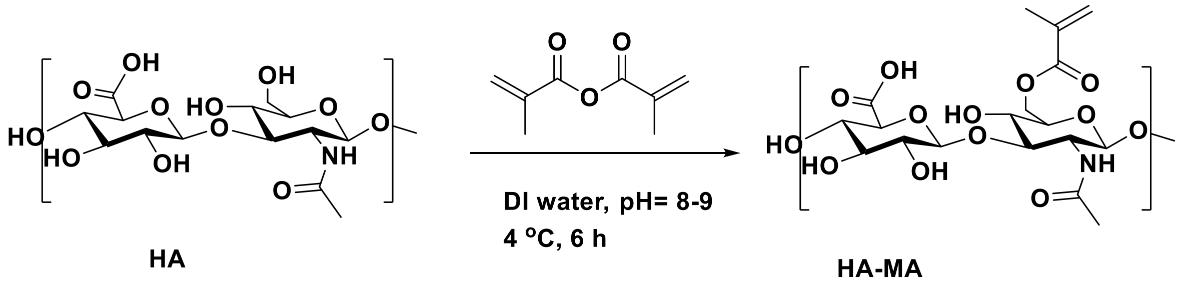

Figure S-1: Synthesis of hyaluronic acid methacrylate (HAMA).

The conjugation of the methacrylate group on hyaluronic acid was confirmed by the presence of distinctive olefinic peaks at 6.08 and 5.69 ppm in the ^{1}H NMR spectrum. The degree of modification was ascertained to be 15 mol% with respect to the repeated disaccharide units of HA. The modification was determined by integrating the olefinic peaks at 6.08 and 5.69 ppm of the methacrylate group against the methyl peak of the N-acetyl of HA at 1.97–2.00 ppm.

2. Synthesis of a hydrazide derivative of gallic acid (GA-Hyd)

The hydrazide derivative of gallic acid was synthesized according to the procedure shown in Figure S-2. In brief, 4 g of GA was dissolved in 100 mL of methanol in a 250 mL flask. Sulfuric acid was added dropwise to the flask, and the reaction mixture was refluxed for 8 hours. After that, the mixture was cooled down to RT, and a rotary evaporator was used to remove the methanol under reduced pressure. The product was then extracted with ethyl acetate, and the organic layer was recovered and dried over anhydrous Na_2SO_4. The solution was dried in a rotary evaporator again to remove the undesired solvent completely. The oily GA methyl ester intermediate was dissolved using 60 mL of methanol, and 2 drops of TEA. 2 mL of 80% hydrazine monohydrate was added to the reaction mixture and stirred for 48 hours at RT. The obtained product was a white solid, which was filtered twice with methanol and again with water. The dried sample was characterized by ^{1}H and ^{13}C NMR spectroscopy, Figure S-4 to Figure S-7.

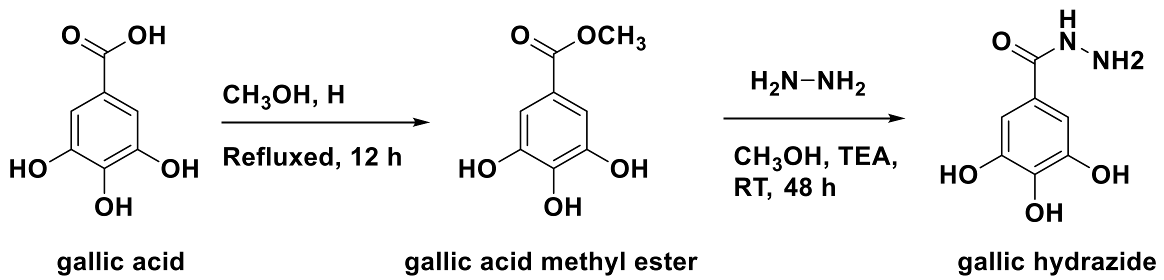

Figure S-2: Synthesis of hydrazide derivative of gallic acid (GA-Hyd).

The pure dried sample was characterized by ^{1}H and ^{13}C NMR spectroscopy. Chemical shifts were measured in δ (ppm) with reference to the DMSO-d6 solvent (δ = 2.50 ppm and 39.92 ppm for ^{1}H and ^{13}C NMR, respectively).

Methyl 3,4,5-trihydroxybenzoate (gallic acid methyl ester): ^{1}H-NMR (500 MHz, DMSO-d6) δ/ppm: 9.27 (s, 2H-OH), 8.93 (s, 1H, -OH), 6.93 (s, 2H, -Ar-H), 3.74 (s, 3H, -O-CH_3). ^{13}C-NMR (125 MHz, DMSO-d6) δ/ppm: 166.76 (-C=O), 146.01 (*m*-Ar-C), 138.84 (*p*-Ar-C), 119.72 (Ar-C-CO-), 108.92 (*o*-Ar-CH), 52.04 (O-CH_3).

3,4,5-trihydroxybenzohydrazide (gallic hydrazide): ^{1}H-NMR (500 MHz, DMSO-d6, D_2O exchange) δ/ppm: 6.09 (s, 2H, Ar-H). ^{13}C-NMR (125 MHz, DMSO-d6) δ/ppm: 165.77 (-C=O), 144.47(*m*-Ar-C), 135.27(*p*-Ar-C), 122.57 (Ar-C-CO-) and 105.59(*o*-Ar-CH).

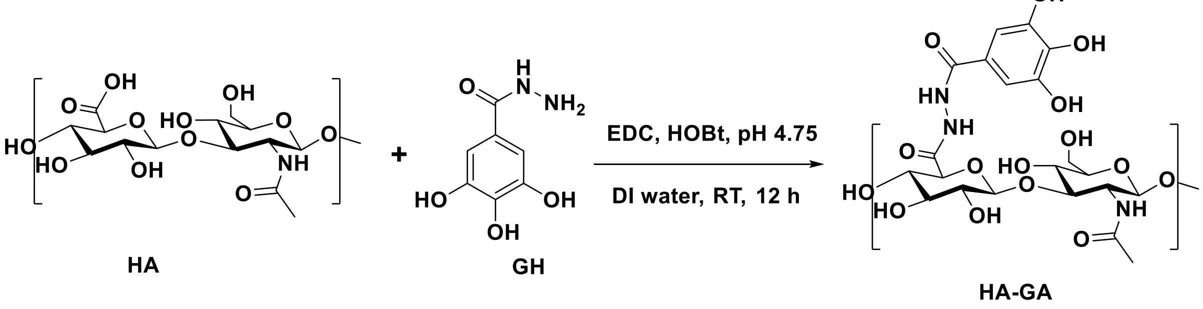

Figure S-3: Synthesis of gallic acid conjugated hyaluronic acid (HAGA).

The conjugation of gallic acid in the hyaluronic acid was confirmed by the presence of distinctive peaks at 6.97 to 6.99 ppm (red) due to aromatic protons of GA in the ^{1}H NMR spectrum.

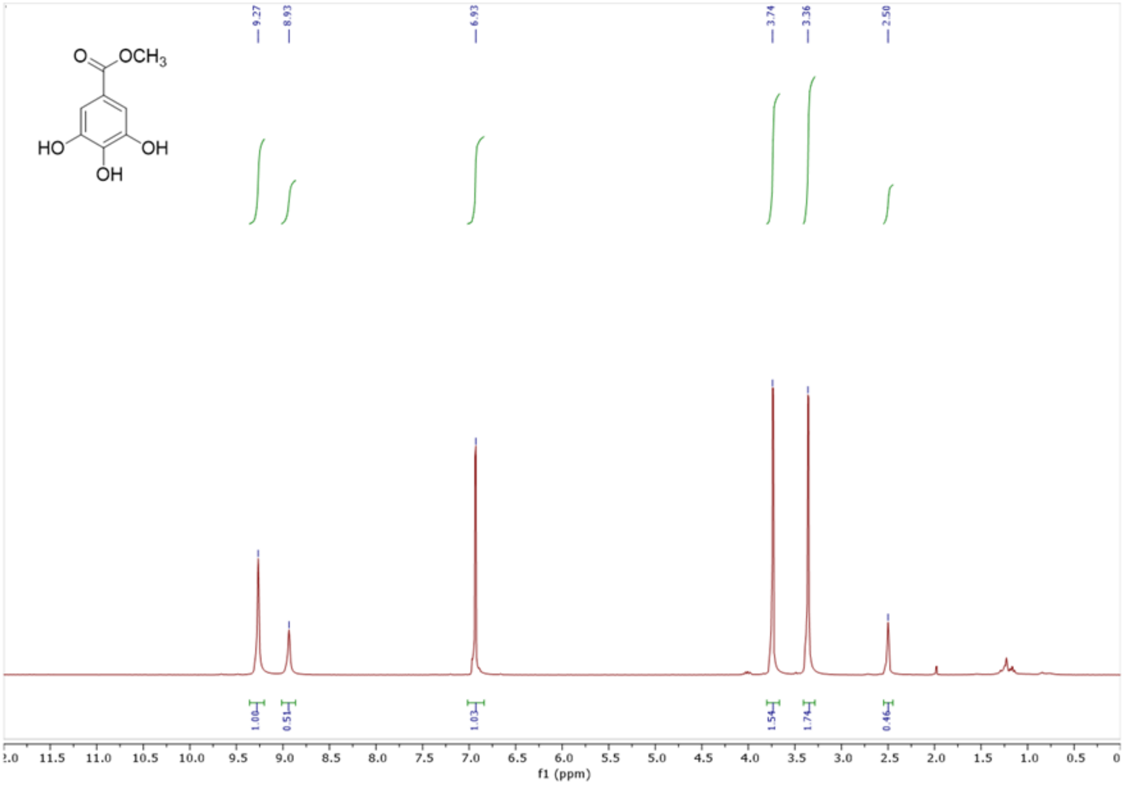

Figure S-4: ^{1}H-NMR (500 MHz) spectrum of gallic acid methyl ester recorded in DMSO-d6.

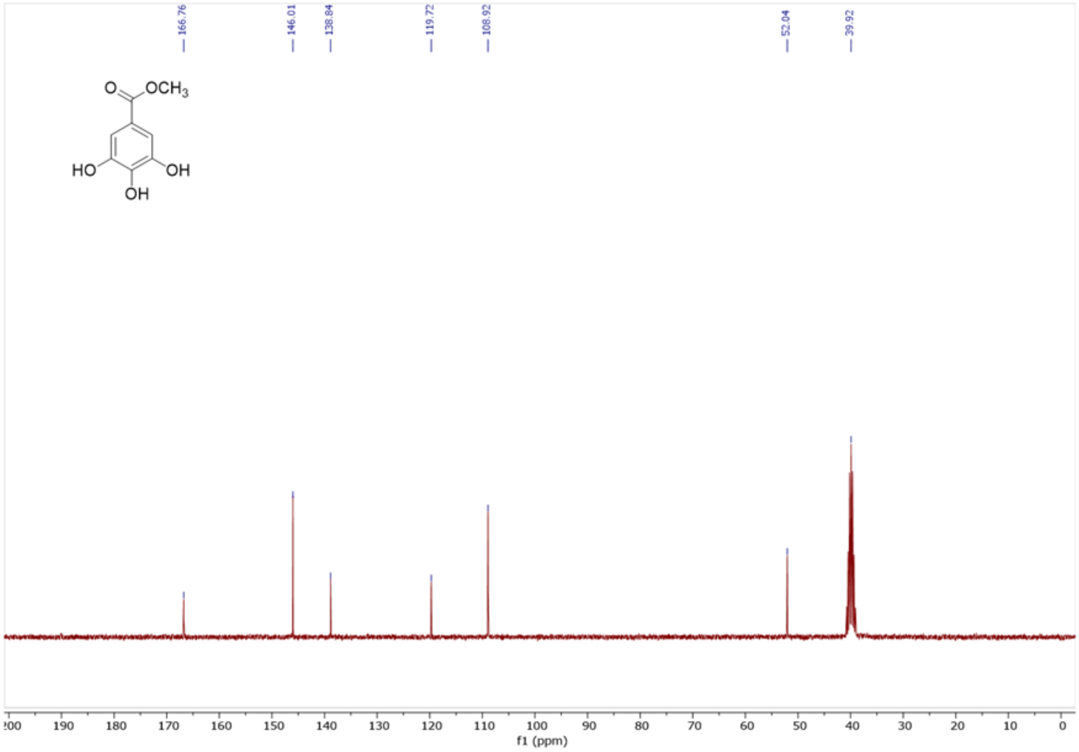

Figure S-5: 13C-NMR (126 MHz) spectrum of gallic acid methyl ester recorded in DMSO-d6.

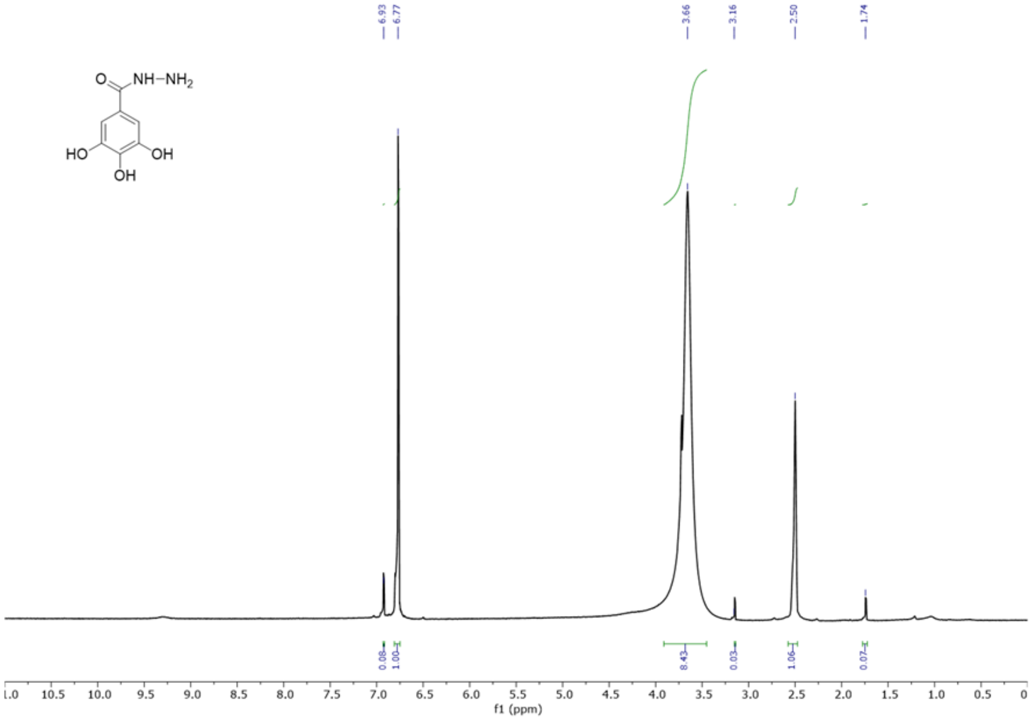

Figure S-6: ^{1}H-NMR (500 MHz) spectra of gallic hydrazide (GA-Hyd) recorded in DMSO-d6.

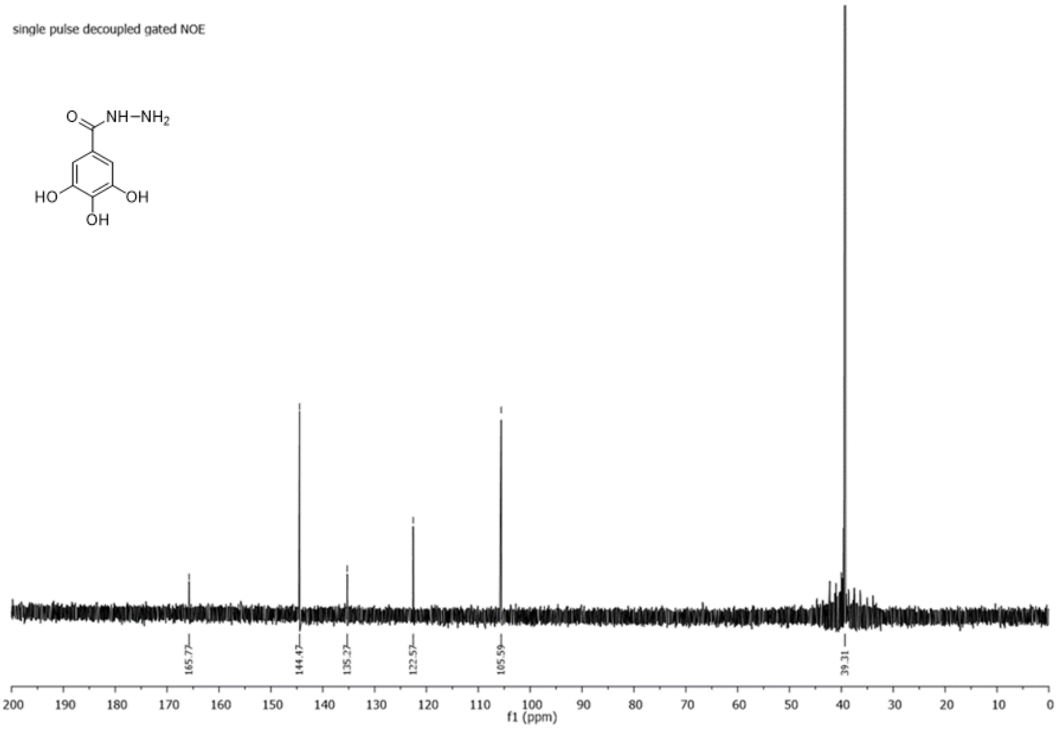

Figure S-7: 13C-NMR (125 MHz) spectra of gallic hydrazide (GA-Hyd) recorded in DMSO-d6.

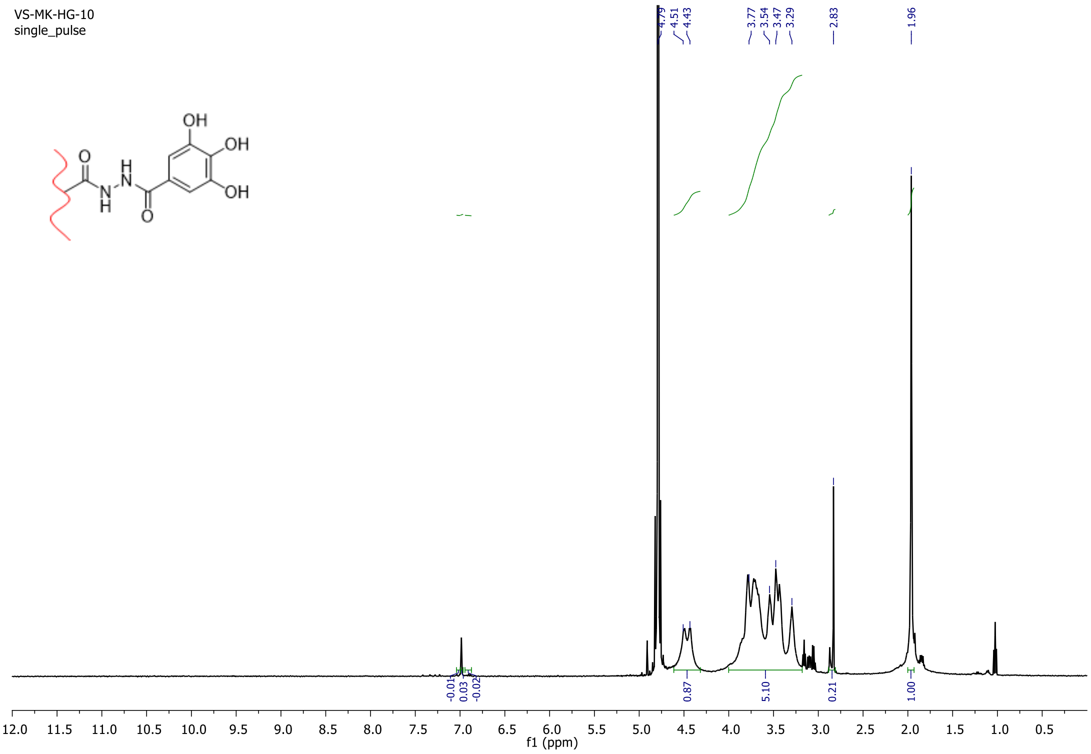

Figure S-8-A: ^{1}H-NMR (500 MHz, D_2O) spectra of GA conjugated HA (HAGA10).

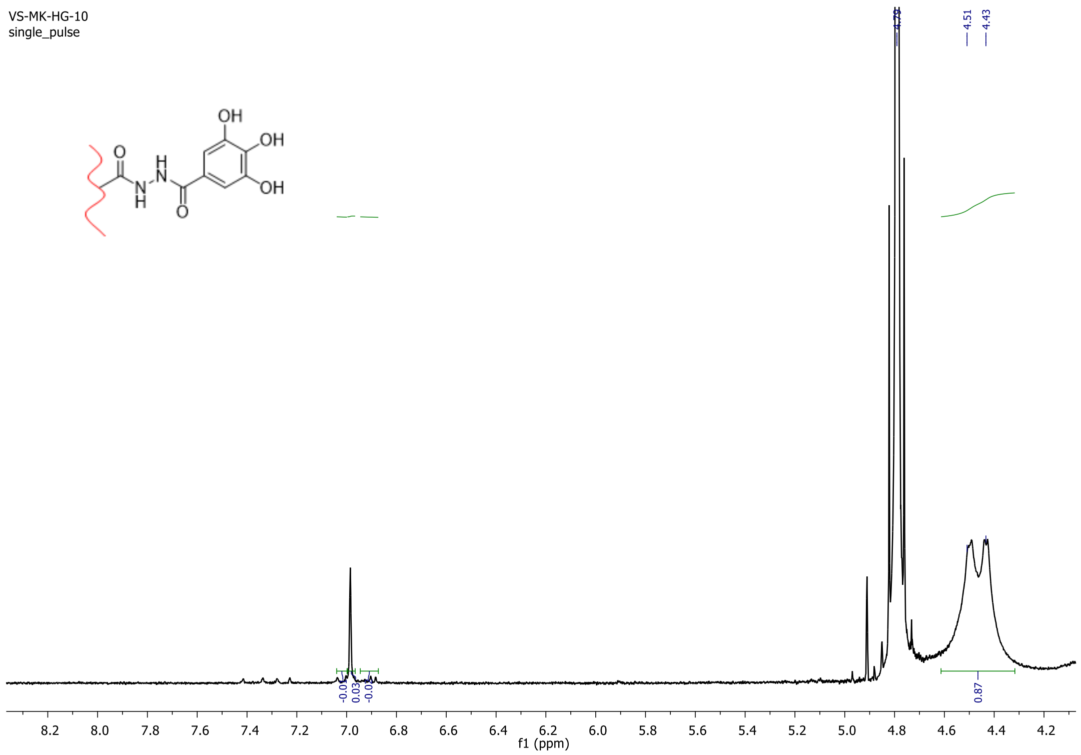

Figure S-8-B: ^{1}H-NMR (500 MHz, D_2O) spectra of GA conjugated HA (HAGA10) (expanded).

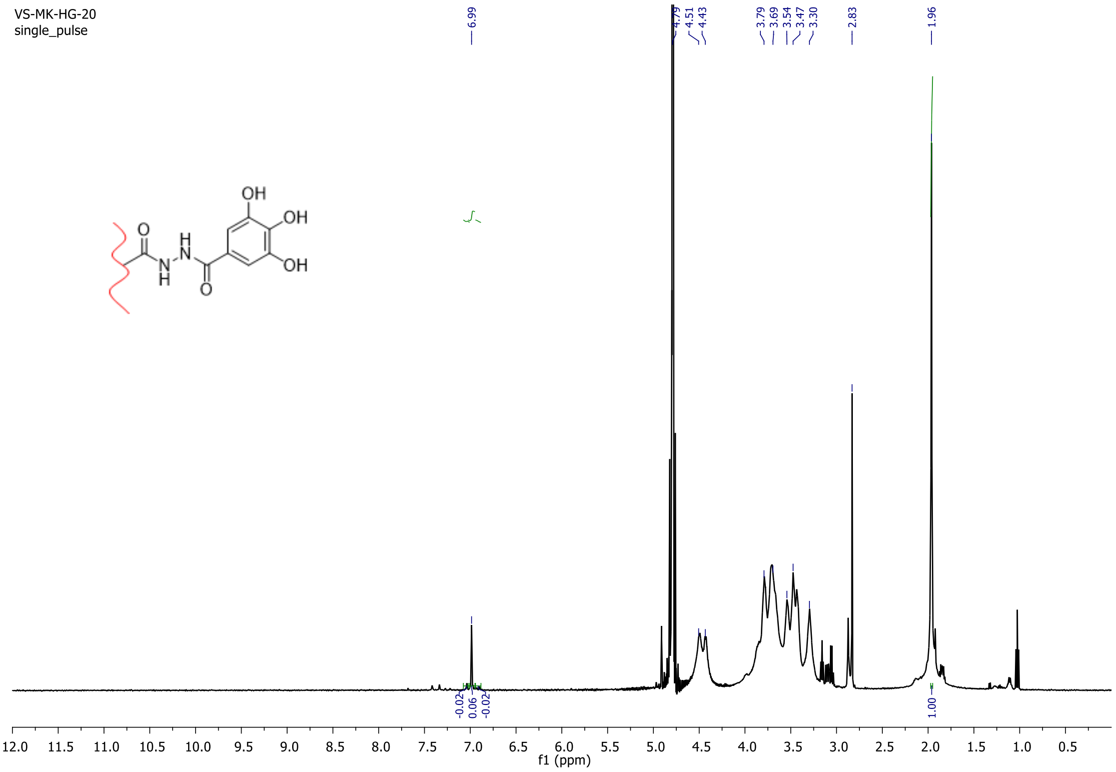

Figure S-9-A: ^{1}H-NMR (500 MHz, D2O) spectra of GA conjugated HA (HAGA20).

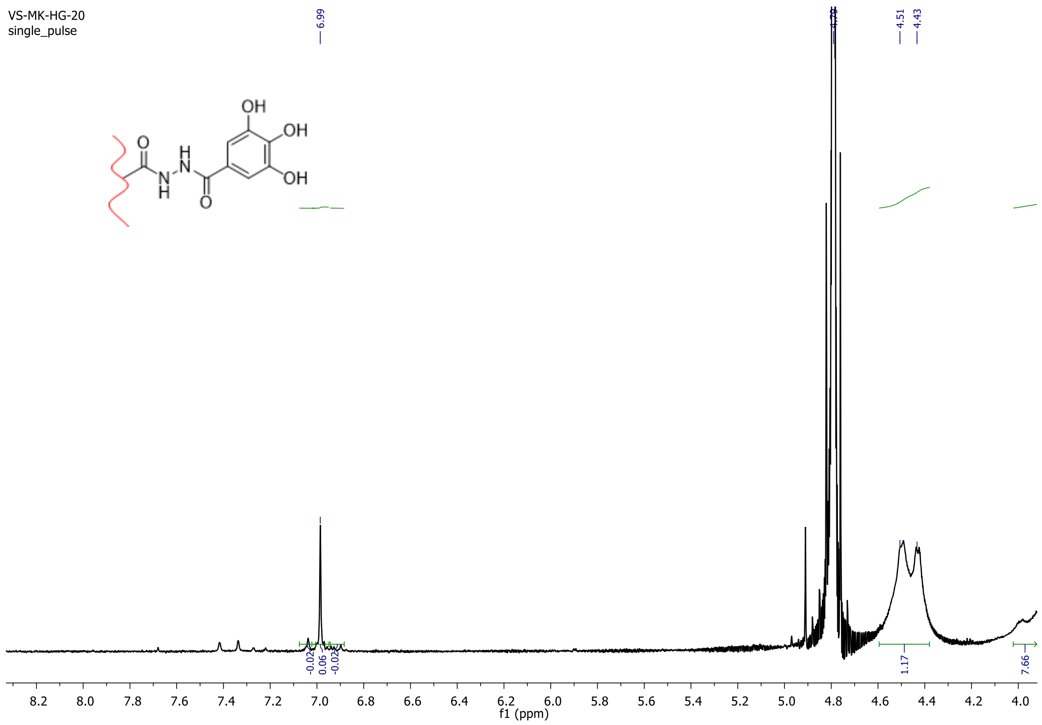

Figure S-9-B: ^{1}H-NMR (500 MHz, D2O) spectra of GA conjugated HA (HAGA20) (expanded).

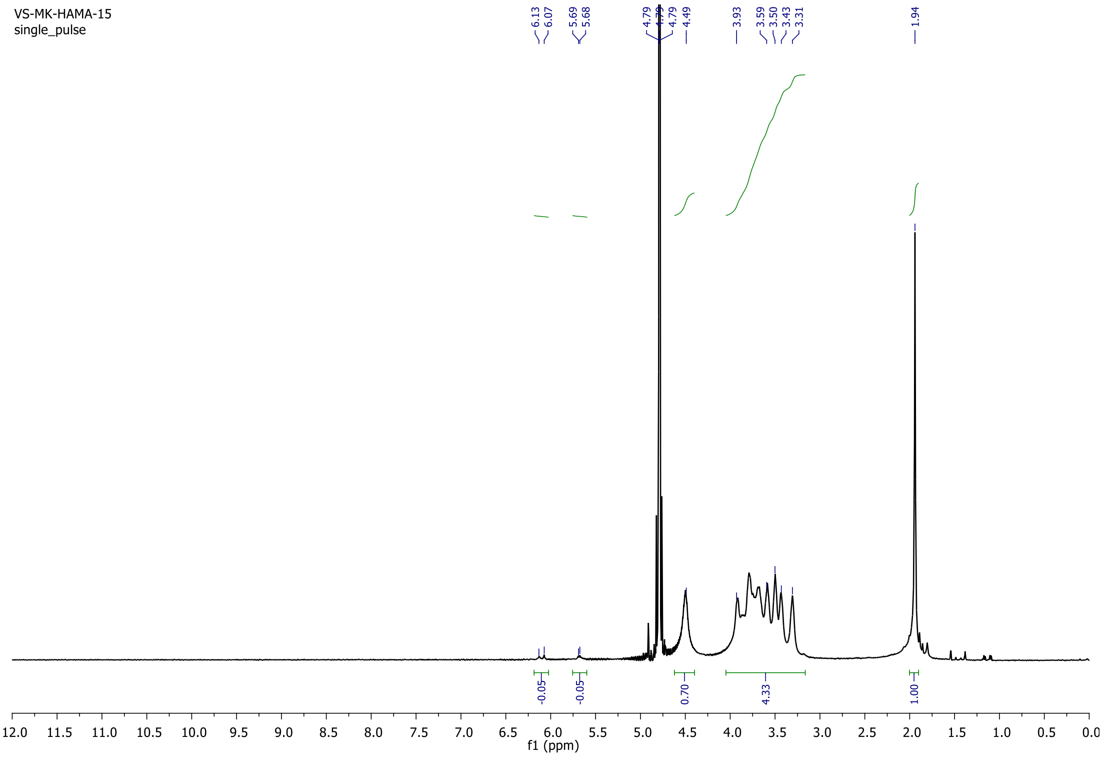

Figure S-10-A: ^{1}H-NMR (500 MHz) spectra of hyaluronic acid methacrylate (15% modification) (HAMA15) recorded in D_2O.

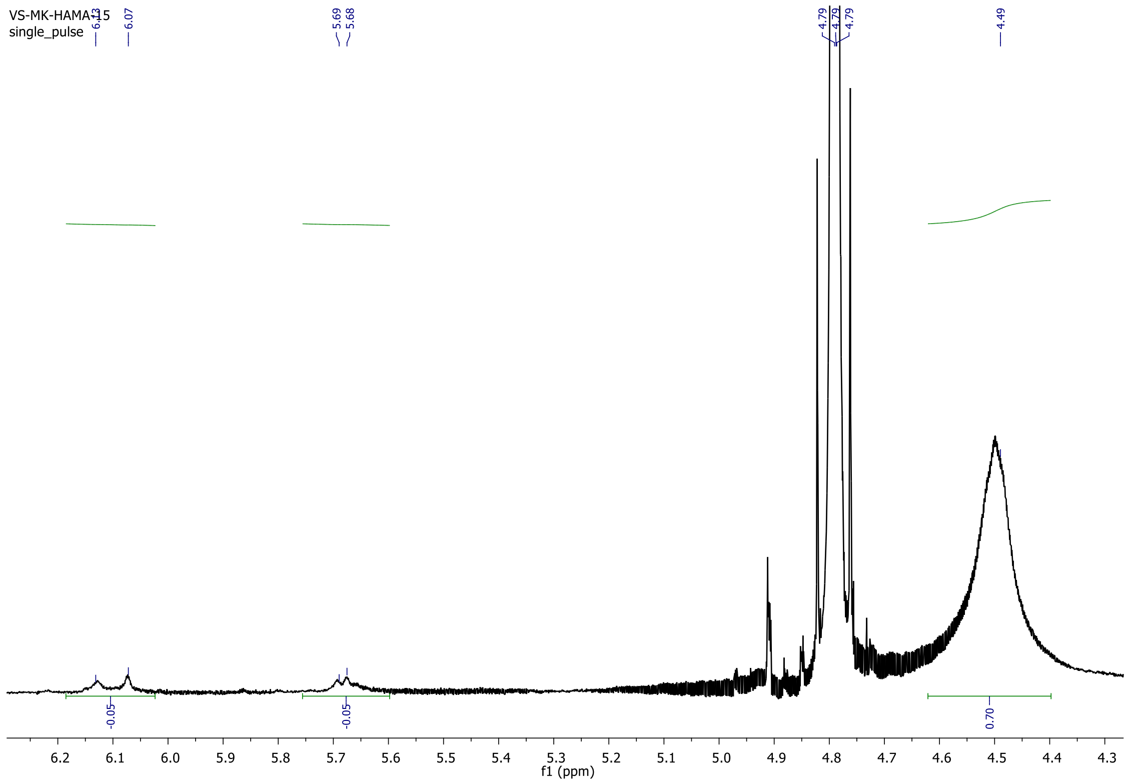

Figure S-10-B: ^{1}H-NMR (500 MHz) spectra of hyaluronic acid methacrylate (15% modification) (HAMA15) recorded in D_2O (expanded).

3. Pr value

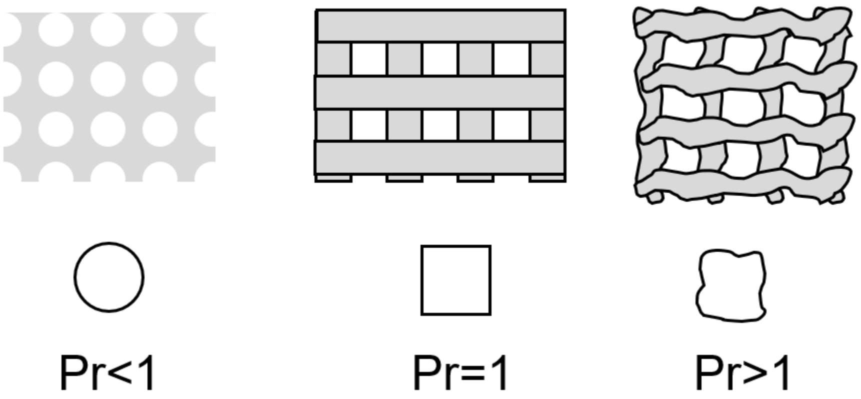

Figure S-11: Pore geometry evaluation and calculation of the printability (Pr) value. Ideally, Pr =1, indicating perfectly square-shaped pores[1,2].

4. Filament quality

The filament quality of plain HAMA15, HAGA10-HAMA15 and HAGA20-HAMA15 was investigated at different pH conditions: at pH 3-5, 7.5-8 and > 8[3].

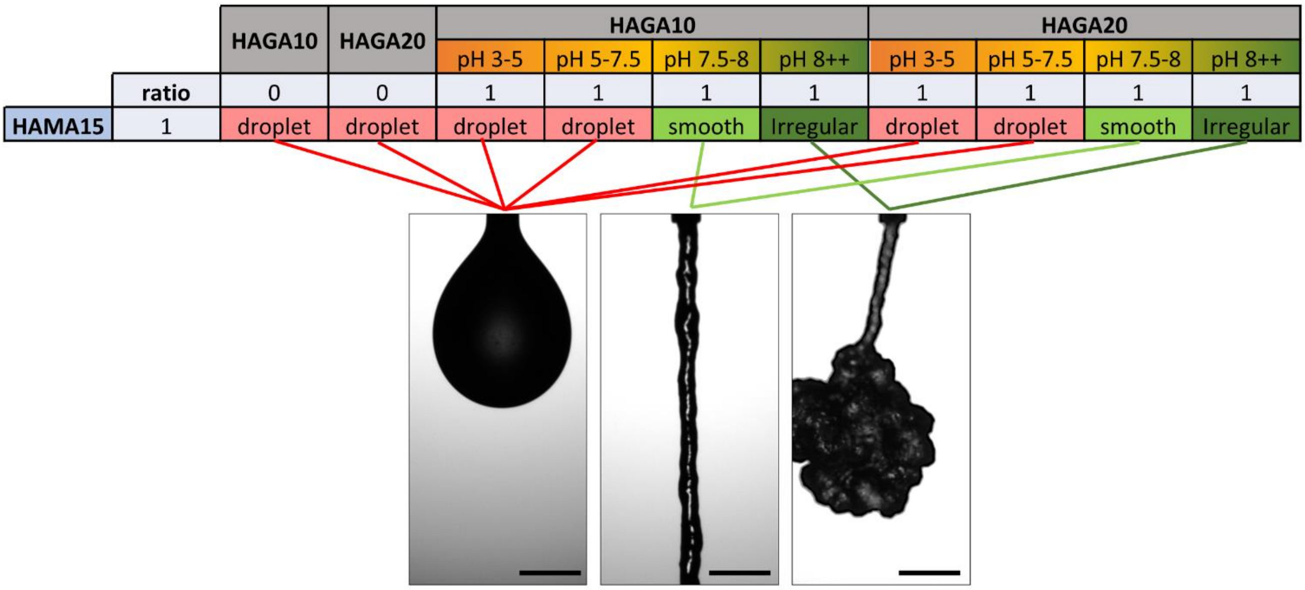

Figure S-12: Filament quality of plain HAMA15, HAGA10-HAMA15 and HAGA20-HAMA15 at pH 3–5, 7.5–8 and > 8 (410 µm nozzle size), 1 mm scale bar.

5. Gelation time[4]

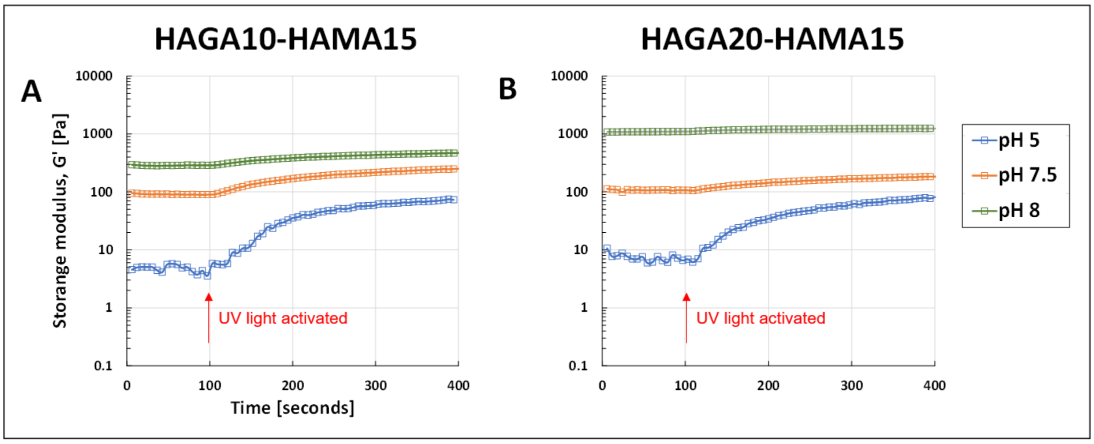

Figure S-13: Gelation time of (A) HAGA10-HAMA15 and (B) HAGA20-HAMA15 after photocrosslinking.

6. Power-Law model (Equation S-1)

$$\mu = K\gamma^{n-1} \quad (1)$$

The flow behavior index n describes the shear-thinning ability of the ink. If n = 1, the ink follows Newtonian behavior. If $n > 0.6$, the material is weakly shear-thinning, and if $n \leq 0.2$, the ink has good shear-thinning properties and excellent printability.

Yield stress values were determined from the yield stress-shear rate plot, where the shear stress begins to increase from the intersection at the Y-axis, using the Herschel-Bulkley model (Equation S-2).

$$\tau = \tau_0 + K\gamma^n, \quad (2)$$

where τ is the shear stress measured on the inks and τ_0 is the yield stress. The yield point determines the flow initiation of the inks at the level of the applied shear stress.

7. Cox-Merz rule

The Cox-Merz rule explains the relationship between the shear viscosity (η) and complex viscosity (η*) from oscillatory measurement, describing the correspondence between the shear viscosity, η, against shear rate, γ, and the complex viscosity (η*) from the oscillatory measurement of frequency sweep (angular frequency, ω) (Equation S-3). Viscosity, as a function of a shear rate based on the Cox-Merz rule, was applied to the oscillatory measurements of frequency sweep and Power-Law fit for the shear thinning region (blue line). The green line indicates the obtained shear-thinning coefficients from the Power-Law model without using the Cox-Merz rule[5].

$$|\eta^*(\omega, \mathrm{rad/s})| = |\eta\ (\gamma, s^{-1})|_{,(\gamma)=(\omega)} \quad (3)$$

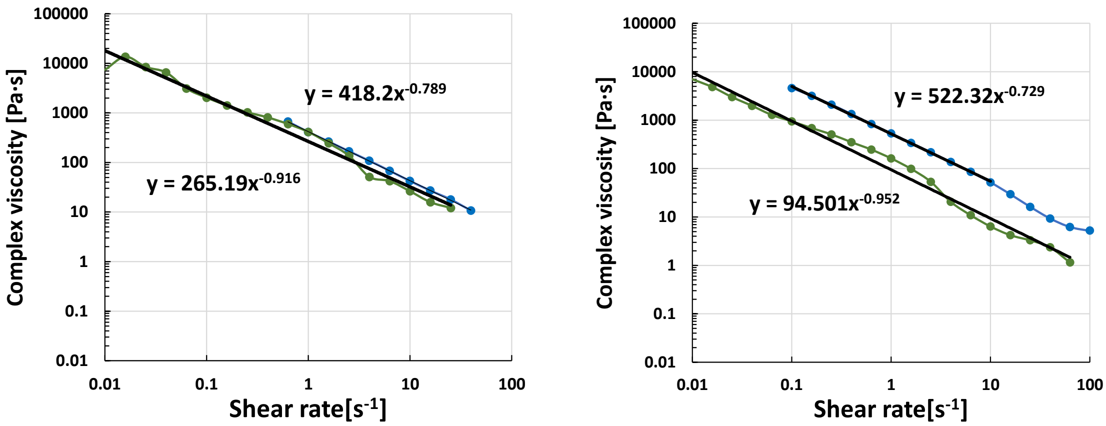

Figure S-14: The comparison of shear-thinning coefficients (HAGA10-HAMA15, left and HAGA20-HAMA15, right) of the Power-Law model with (blue line) and without (green line) using the Cox-Mertz rule.

Table S-1: Values of shear-thinning coefficients. The precursors with shear-thinning coefficients in bold were used to screen true printability. (xx*) = *n* values calculated via the Cox-Mertz rule.

**Precursors (at 37 °C)**	***n***	τ_0
**HAGA10-HAMA15 at pH 3**	0.92	0.06
**HAGA10-HAMA15 at pH 5.5**	0.97	0.06
**HAGA10-HAMA15 at pH 7.5**	0.23	135
**HAGA10-HAMA15 at pH 8**	**0.22**	**110**
**HAGA10-HAMA15 at pH 9**	**n/a (0.21*)**	**215**
**HAGA20-HAMA15 at pH 3**	0.95	0.05
**HAGA20-HAMA15 at pH 5.5**	0.96	0.07
**HAGA20-HAMA15 at pH 7.5**	0.18	21
**HAGA20-HAMA15 at pH 8**	0.21	209

**HAGA20-HAMA15 at pH 9**		**0.05 (0.27*)**	**460**

8. Plain HAMA15 and HAGA20-HAMA15 (neutral pH) printing test.

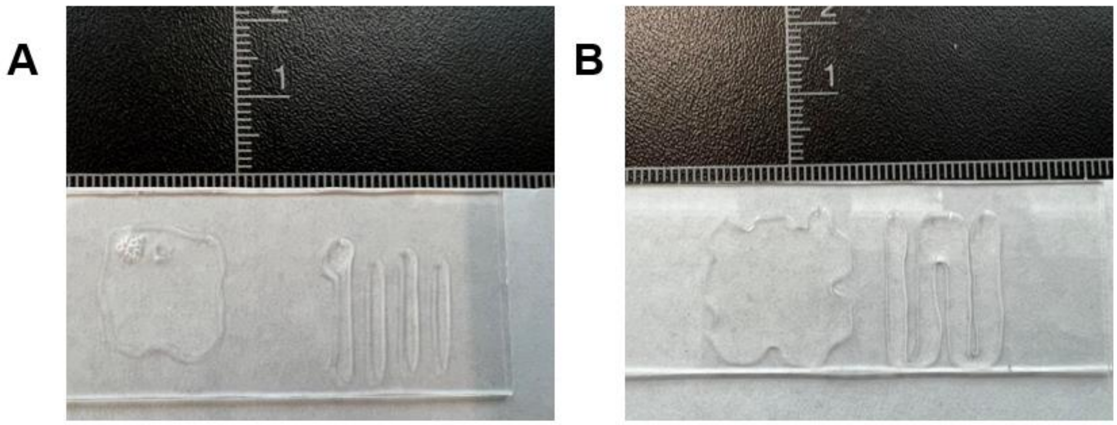

Figure S-15: (A) The printed filaments of HAMA confirmed the poor printability, as they exhibited liquid-like filaments and were not able to maintain shape fidelity. (Left) The grid structures failed to form, and the filaments merged together. (Right) The deposited filaments started to spread immediately after contacting the glass substrate. (B) HAGA10-HAMA15 and HAGA20-HAMA15 printouts at the neutral pH. The ruler scale is 1 cm.

9. Pr value equation

As described in the main article, biomaterial inks with proper rheological properties should produce a filament with a smooth surface and coherent width while being able to stack without merging. Bioinks with excellent printability will provide high resolution, exhibiting constant shape and square pores. The printability assessment of different biomaterial ink compositions was done by printing two-layered grid patterns. The shape of the printed pores was evaluated using Equation S-4[6,7].

$$Pr = \frac{\pi}{4} \cdot \frac{1}{C} = \frac{L^2}{16A}, (4)$$

in which C is the circularity of the enclosed pore, L is the perimeter and A the pore area. The printability (Pr) of the biomaterial ink compositions was determined by the squareness of the pores inside the grid structure. Pr value 1 indicates a perfect square shape.

A B C

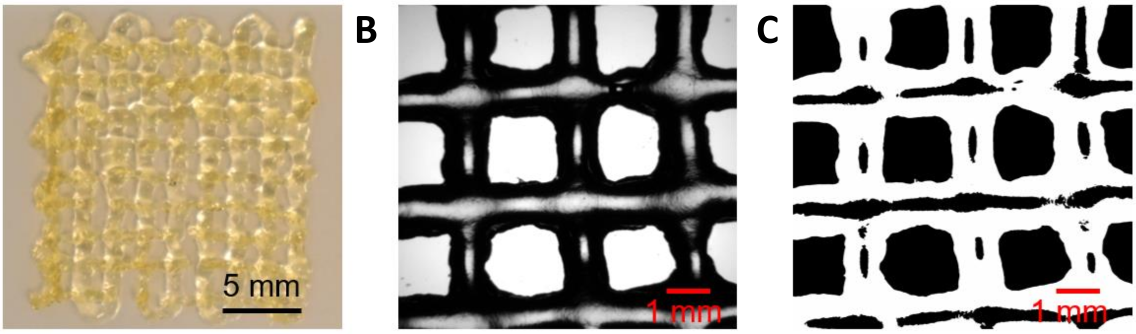

Figure S-16: An example of biomaterial ink printability assessment from the macrostructure of HAGA20-HAMA15 at pH 7.5–8. The Pr values were calculated from the average squareness of the pores inside the printed grid structure. (A) Photo of a printed grid structure of HAGA20-HAMA15, (B) optical microscope image of HAGA20-HAMA15, and (C) the processed image (ImageJ) for Pr value calculation.

Table S-2: Pr values of printed grid structures in Figure S-16.

Precursors	Pr value
**HAGA20-HAMA15 (small grids)**	1.08 ± 0.14
**HAGA20-HAMA15 (large grids)**	0.99 ± 0.10

10. 3D printed cylinder structures of HAGA20-HAMA15 at pH 7.5–8

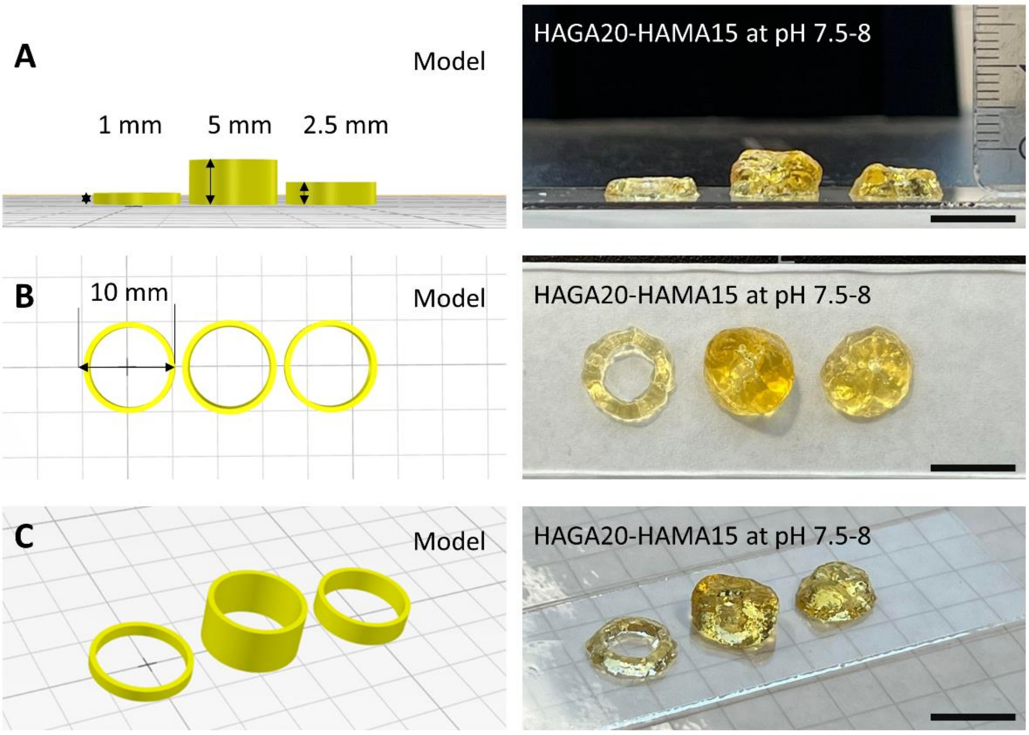

Figure S-17: The images of 3D printed cylinders (HAGA20-HAMA15 at pH 7.5–8), varying the height: 1, 2.5 and 5 mm, compared to the CAD model. (A) Side view, (B) top view, and (C) isometric view. Scale bar = 10 mm.

11. Crosslinking density and average mesh size

For further in-depth structural analysis, the average mesh size and crosslinking density were determined from rheological measurement results. The average mesh size (ξ, nm) calculation was applied using the storage moduli (*G'*) of resulting hydrogels (the best formulation ink) at 120 s UV exposure time[8]. Equation S-5 estimates the average mesh size (ξ) of hydrogels at different exposure times:

$$(\xi) = \left(\frac{G'N}{RT}\right)^{-1/3}, (5)$$

where G' is the storage modulus of the hydrogel, N is the Avogadro constant (6.023×10^{23} mol^{-1}), R is the molar gas constant (8.314 $JK^{-1}mol^{-1}$), and T is the temperature (298 K).

Moreover, crosslinking density (n_e, mol/m^3) of the hydrogels was calculated using the storage modulus from the linear region of the frequency sweep test. The data provided the total number of elastically active junction points in the network per unit of volume, using Equation S-6[8].

$$n_e = \frac{G_e}{RT}, (6)$$

where G_e is the average value of storage modulus from the linear region of oscillatory frequency sweep measurement.

Table S-3 Mesh size and crosslinking density of hydrogels.

	Mesh size	Crosslinking density
HAGA10-HAMA15 w/o UV	19 nm	0.22
HAGA10-HAMA15 with UV	19 nm	0.24
HAGA20-HAMA15 w/o UV	17 nm	0.33
HAGA20-HAMA15 with UV	16 nm	0.40

12. Self-healing behavior

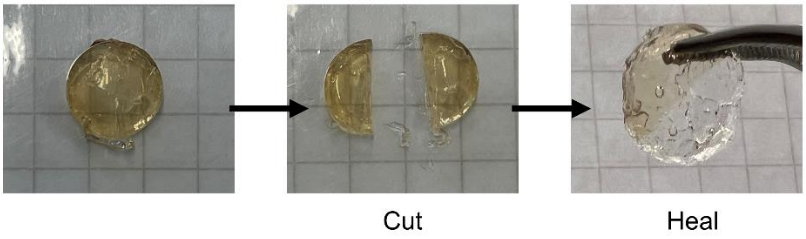

Figure S-18: The figure demonstrates the mild self-healing behavior of HAGA20-HAMA15 hydrogels[9,10].

13. Degradation study

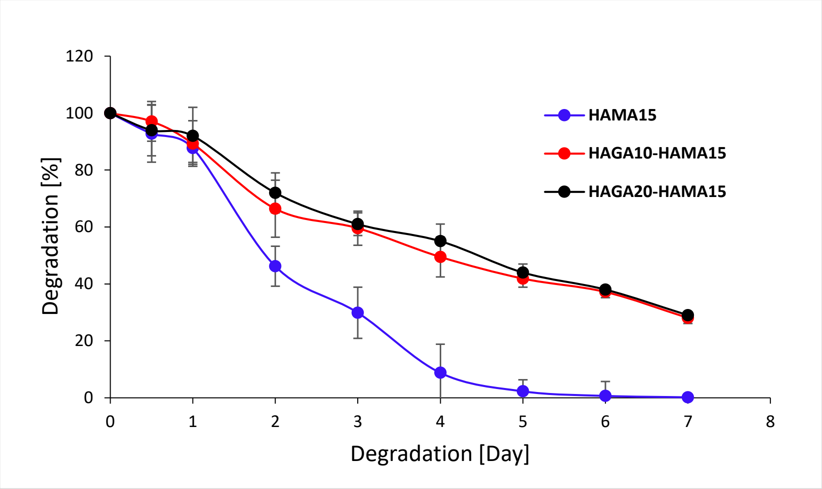

Figure S-19: The enzymatic degradation study of HAMA15, HAGA10-HAMA15 and HAGA20-HAMA15 in hyaluronidase-contained DPBS at 37 °C.

Reference

(1) Jongprasitkul, H.; Turunen, S.; Parihar, V. S.; Kellomäki, M. Two-Step Crosslinking to Enhance the Printability of Methacrylated Gellan Gum Biomaterial Ink for Extrusion-Based 3D Bioprinting. *Bioprinting* **2022**, *25*. https://doi.org/10.1016/j.bprint.2021.e00185.

(2) Jongprasitkul, H.; Turunen, S.; Parihar, V. S.; Kellomäki, M. Sequential Cross-Linking of Gallic Acid-Functionalized GelMA-Based Bioinks with Enhanced Printability for Extrusion-Based 3D Bioprinting. *Biomacromolecules* **2023**, *24* (1), 502–514. https://doi.org/10.1021/acs.biomac.2c01418.

(3) Paxton, N.; Smolan, W.; Böck, T.; Melchels, F.; Groll, J.; Jungst, T. Proposal to Assess Printability of Bioinks for Extrusion-Based Bioprinting and Evaluation of Rheological Properties Governing Bioprintability. *Biofabrication* **2017**, *9* (4), 44107. https://doi.org/10.1088/1758-5090/aa8dd8.

(4) O'Connell, C. D.; Zhang, B.; Onofrillo, C.; Duchi, S.; Blanchard, R.; Quigley, A.; Bourke, J.; Gambhir, S.; Kapsa, R.; Di Bella, C.; Choong, P.; Wallace, G. G. Tailoring the Mechanical Properties of Gelatin Methacryloyl Hydrogels through Manipulation of the Photocrosslinking Conditions. *Soft Matter* **2018**, *14* (11), 2142–2151. https://doi.org/10.1039/c7sm02187a.

(5) Włodarczyk-Biegun, M. K.; Paez, J. I.; Villiou, M.; Feng, J.; Del Campo, A. Printability Study of Metal Ion Crosslinked PEG-Catechol Based Inks. *Biofabrication* **2020**, *12* (3), 035009. https://doi.org/10.1088/1758-5090/ab673a.

(6) Ouyang, L.; Armstrong, J. P. K.; Lin, Y.; Wojciechowski, J. P.; Lee-Reeves, C.; Hachim, D.; Zhou, K.; Burdick, J. A.; Stevens, M. M. Expanding and Optimizing 3D Bioprinting Capabilities Using Complementary Network Bioinks. *Sci Adv* **2020**, *6* (38), 1–14. https://doi.org/10.1126/sciadv.abc5529.

(7) Gillispie, G.; Prim, P.; Copus, J.; Fisher, J.; Mikos, A. G.; Yoo, J. J.; Atala, A.; Lee, S. J. Assessment Methodologies for Extrusion-Based Bioink Printability. *Biofabrication* **2020**, *12* (2), 022003. https://doi.org/10.1088/1758-5090/ab6f0d.

(8) Karvinen, J.; Ihalainen, T. O.; Calejo, M. T.; Jönkkäri, I.; Kellomäki, M. Characterization of the Microstructure of Hydrazone Crosslinked Polysaccharide-Based Hydrogels through Rheological and Diffusion Studies. *Materials Science and Engineering C* **2019**, *94* (January 2019), 1056–1066. https://doi.org/10.1016/j.msec.2018.10.048.

(9) Han, L.; Yan, L.; Wang, K.; Fang, L.; Zhang, H.; Tang, Y.; Ding, Y.; Weng, L. T.; Xu, J.; Weng, J.; Liu, Y.; Ren, F.; Lu, X. Tough, Self-Healable and Tissue-Adhesive Hydrogel with Tunable Multifunctionality. *NPG Asia Materials 2017 9:4* **2017**, *9* (4), e372–e372. https://doi.org/10.1038/am.2017.33.

(10) Bertsch, P.; Diba, M.; Mooney, D. J.; Leeuwenburgh, S. C. G. Self-Healing Injectable Hydrogels for Tissue Regeneration. *Chemical Reviews*. American Chemical Society January 25, 2023, pp 834–873. https://doi.org/10.1021/acs.chemrev.2c00179.
